# Supplementary material for: Developmental transformations of Purkinje cells tracked by DNA electrokinetic mobility
Source: Cell Rep Methods. 2025 Aug 26;5(9):101143. doi: 10.1016/j.crmeth.2025.101143 (PMC12539252; doi:10.1016/j.crmeth.2025.101143)
Supplement: Document S2. Article plus supplemental information [file mmc5.pdf]

Cell Reports
Methods

Article

Developmental transformations of Purkinje cells tracked by DNA electrokinetic mobility

Graphical abstract

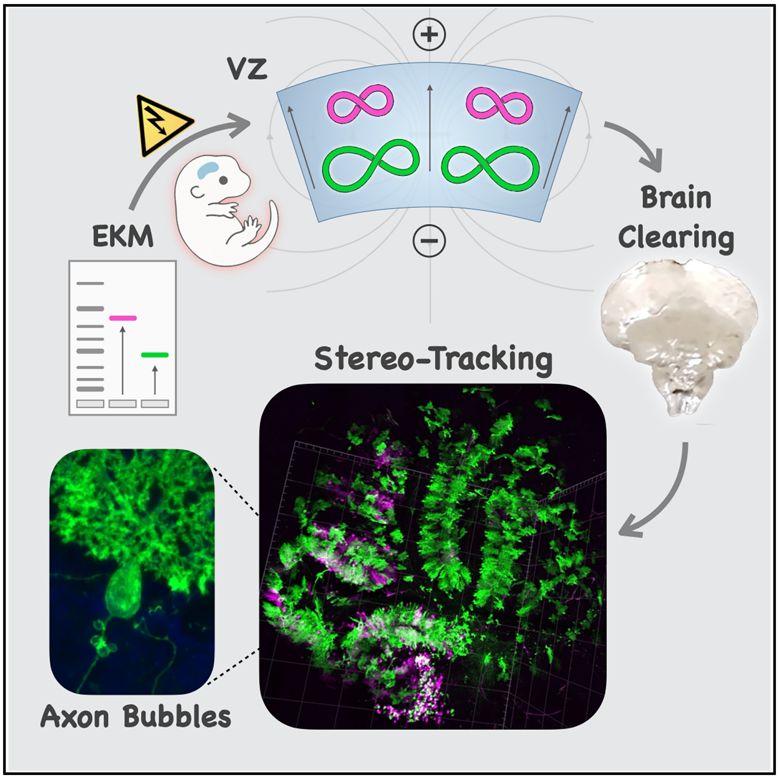

Highlights

- DNA size and electrokinetic mobility determine electroporation depth in neural tissue
- Stereo-tracking uses DNA mobility to label progenitor zones at distinct depths
- Axon bubbles appear on Purkinje cell axon initial segments during late development
- Progenitor zone subfields map to Purkinje cell topography in the cerebellar cortex

Authors

Cheryl Brandenburg, Garrett W. Crutcher, Andrea J. Romanowski, ..., Gene J. Blatt, Roy V. Sillitoe, Alexandros Poulopoulos

Correspondence

brandenb.cheryl@gmail.com (C.B.), apoulopoulos@som.umaryland.edu (A.P.)

In brief

By harnessing the bioelectric properties of DNA, Brandenburg et al. develop "stereo-tracking," a method to label neural progenitors based on depth within the ventricular zone. This enables 3D mapping of neuronal development *in vivo*, uncovering remarkable developmental trajectories and unexpected axonal features in Purkinje cells.

Brandenburg et al., 2025, Cell Reports Methods *5*, 101143
September 15, 2025 © 2025 The Author(s). Published by Elsevier Inc.
https://doi.org/10.1016/j.crmeth.2025.101143

CellPress

Cell Reports Methods

CellPress
OPEN ACCESS

Article

Developmental transformations of Purkinje cells tracked by DNA electrokinetic mobility

Cheryl Brandenburg,[1,2,*] Garrett W. Crutcher,[1] Andrea J. Romanowski,[1] Sarah G. Donofrio,[2] Lita R. Duraine,[2] Richard N.A. Owusu-Mensah,[3,7] Benjamin H. Cooper,[4] Izumi Sugihara,[3,5] Gene J. Blatt,[6] Roy V. Sillitoe,[2] and Alexandros Poulopoulos[1,8,*]

[1]Department of Pharmacology and Physiology, University of Maryland School of Medicine, Baltimore, MD, USA
[2]Department of Pathology & Immunology, Baylor College of Medicine, Jan and Dan Duncan Neurological Research Institute, Texas Children's Hospital, Houston, TX, USA
[3]Department of Systems Neurophysiology, Graduate School of Medical and Dental Sciences, Tokyo Medical and Dental University, Bunkyo-Ku, Tokyo, Japan
[4]Department of Molecular Neurobiology, Max Planck Institute for Multidisciplinary Sciences, Göttingen, Germany
[5]Department of Health Science, Faculty of Sports & Health Science, Daito Bunka University, Higashimatsuyama-shi, Saitama, Japan
[6]Graduate Program in Life Sciences, University of Maryland School of Medicine, Baltimore, MD, USA
[7]Present address: Department of Molecular Pharmacology and Experimental Therapeutics, Mayo Clinic, Rochester, MN, USA
[8]Lead contact
*Correspondence: brandenb.cheryl@gmail.com (C.B.), apoulopoulos@som.umaryland.edu (A.P.)
https://doi.org/10.1016/j.crmeth.2025.101143

MOTIVATION Understanding how progenitor zone organization determines the complex topography of mature neural circuits remains challenging, particularly in regions like the cerebellum, where developmental trajectories are intricate. Current methods for tracking neuronal migration typically rely on birthdate labeling or lineage tracing, but lack the ability to simultaneously distinguish cells from different spatial positions within 3D progenitor fields. This limitation hinders our ability to map how spatial organization in embryonic zones translates to the stereotyped patterns observed in adult brain circuitry. A method enabling differential labeling based on progenitor zone depth would provide critical insights into developmental patterning principles.

SUMMARY

Brain development begins with neurogenesis in progenitor zones and ends with expansive, intricately-patterned cellular diversity in the adult brain. We took advantage of bioelectric interactions between DNA and embryonic tissue to perform "stereo-tracking," a developmental targeting strategy that differentially labels cells at different depths within progenitor zones. This 3D labeling was achieved by delivery of plasmids with distinct electrokinetic mobilities *in utero*. We applied stereo-tracking with light sheet imaging in the cerebellum and identified that Purkinje cells follow embryonically committed developmental trajectories, linking distinct progenitor zone subfields to the mature topography of the cerebellar cortex. We additionally identified an unexpected subcellular structure on the axon initial segment of Purkinje cells that we termed "axon bubbles." These structures were revealed by glycosylphosphatidylinositol (GPI)-linked surface labeling and confirmed by electron microscopy. Our findings demonstrate organization of neural progenitor zones in three dimensions, exemplifying the potential of stereo-tracking to uncover new biology within developing systems.

INTRODUCTION

Development of the central nervous system (CNS) unfolds through a complex orchestration of cellular transformations, from the progenitor zones of mitotic cells to the gray matter positions of postmitotic neurons and the white matter tracks of their axon projections, to form the intricate connectivity of mature neural circuitry. Tracking the stereotyped patterns of these transformations is an important aspect of developmental neuroscience and is enabled by powerful molecular methods, such as *in utero* DNA electroporation[1] and lineage tracing.[2] Despite these advances, brain areas with complex patterning, like the cerebellum, still present significant challenges in revealing their developmental programs.

© 2025 The Author(s). Published by Elsevier Inc.
This is an open access article under the CC BY license (http://creativecommons.org/licenses/by/4.0/).

Cerebellar Purkinje cells, central to cerebellar circuits, have particularly complex development, with convoluted migration trajectories that form clusters before dispersal into the expanding foliated lobules.[3–5] Despite this complexity, their ultimate placement within the mature cerebellum appears to be determined at neuron birth.[6–8]

Here, we identify a bioelectric interaction between DNA and neural progenitor zones, which we leverage to develop a method we call "stereo-tracking" to track migratory trajectories from progenitor zone subfields to gray matter cell body positions and white matter topography of axon projections. We apply stereo-tracking to the mouse developing cerebral and cerebellar cortices and analyze migration and topographic positioning in sections and intact brains in 3D. Through the labeling modalities of this method, we additionally uncovered subcellular axonal structures that emerge during Purkinje cell development, which we term "axon bubbles." Finally, we provide technical details and resources for investigations using a labeling toolkit for progenitor zone stereo-tracking and 3D analysis.

RESULTS

Differential plasmid DNA expression based on bioelectric interactions *in utero*

We observed that mixtures of plasmids electroporated *in utero* can be expressed in distinct cell populations, even though the expression was driven by the same promoter. We investigated the circumstances under which plasmids from a single electroporation (Figure 1A) segregate into distinct neurons by testing different combinations of plasmids ubiquitously expressing green and red fluorescent protein (FP) variants, which we term GFP and RFP, respectively, for simplicity (see STAR Methods and Figure S1). We determined that segregation of mixed plasmids into distinct postmitotic neurons was not driven by promoter or plasmid sequence but rather was an electrogenic effect correlating with the relative electrokinetic mobility (EKM) of the plasmids in the mixture. Similar to how EKM determines plasmid electrophoretic mobility and separation of DNA mixes on a gel according to molecular weight and supercoiled state[9,10] (Figure S2), we observed plasmid expression segregating into distinct neuron populations according to each plasmid's EKM (Figures 1B–1E).

When two plasmids of equal EKM (same size and supercoiled state) were co-electroporated into the dorsal pallium progenitor zone, they co-expressed in the same neurons (Figure 1D). However, when electrokinetically faster and slower plasmids (lower and higher molecular weights, respectively) were co-electroporated, faster plasmids additionally expressed in a fraction of neurons that did not receive slower plasmids. Neurons expressing only fast plasmid were consistently farther along in radial migration at embryonic day 15.5 (E15.5) (Figure 1B, b') and resided in deeper layers at postnatal day 7 (P7) compared to neurons receiving both fast and slow plasmids (Figure 1C). Given the inside-out progression of development in the cerebral cortex,[11,12] these data indicate that fast plasmids reached an earlier-born population of neurons, which had a head start in radial migration, and ended up in deeper layers of the cortical plate.

We interpret these observations as follows: the electroporation pulse at E14.5 caused a bioelectric interaction in which each plasmid traveled a distance within the progenitor zone proportional to its EKM. Within the given duration of the electric pulse, fast plasmids (smaller and/or supercoiled) penetrate deeper into the progenitor zone compared to slow plasmids (larger and/or relaxed), much like uncut plasmid migration in gel electrophoresis (Figure S2). Thus, the faster plasmid of the mixture reaches deeper subfields within the progenitor zone, targeting older cells that are farther along in the developmental progression from neurogenesis to radial migration.

The gestational day of targeting determines the precise cell populations that are labeled—in this case, cells destined to become upper-layer glutamatergic neurons. We cannot discern the precise stage in the continuum of mitosis, interkinetic nuclear migration, and early radial migration in which plasmids are received; however, there is a range that our approach bisects: slow plasmids target progenitors in the shallowest layers of the ventricular surface that correspond to labeled postmitotic neurons in the most superficial layers of the cortical plate, while fast plasmids target older cells on their way into radial migration that correspond to labeled postmitotic neurons in deeper layers. This range appears to be analog, since the physical separation of plasmid expression between the layers of cortex, as well as the intensity of fluorescence, scale depending on the EKM difference between the two plasmids and their titrated concentrations (Figure S3).

Manipulation of DNA electrokinetic mobility enables stereo-tracking of neural development

We leveraged the differential targeting of daughter-neuron populations with a single electroporation pulse using plasmids of distinct EKMs to establish an *in utero* approach for differential labeling of cells that are shallower vs. deeper into ventricular progenitor zones. We term this approach "stereo-tracking" as it enables simultaneous labeling of cells at distinct depths within a three-dimensional progenitor zone.

In the radially organized progenitor zone of the dorsal pallium, shallow- vs. deep-labeled cells correspond to younger vs. older, respectively, postmitotic neurons. During the ensuing "chase" period after electroporation, the trajectories of two generations of daughter neurons from the same progenitor zone can be tracked to their distinct layer positions in the cortical plate (Figures 1 and S3).

Stereo-tracking can be viewed as a developmentally orthogonal labeling strategy to lineage tracing.[2] While lineage tracing vertically labels all generations of daughter cells from individual progenitors, stereo-tracking labels a snapshot of two populations based on their location within the progenitor zone. This includes labeling younger vs. older generations, most prominent in simple progenitor zones, as well as convolutions of internal 3D topography in more complex progenitor zones, as in the example in the next section (Stereo-tracking in the cerebellum reveals topographic migration of Purkinje cells).

To facilitate labeling clarity of younger vs. older batches of postmitotic neurons, we employed a binary labeling strategy based on Cre recombinase, similar to that previously employed for *in utero* mosaic manipulation approaches.[13–15] The fast plasmid encodes floxed-RFP/stop/GFP, and the slower plasmid

CellPress
OPEN ACCESS

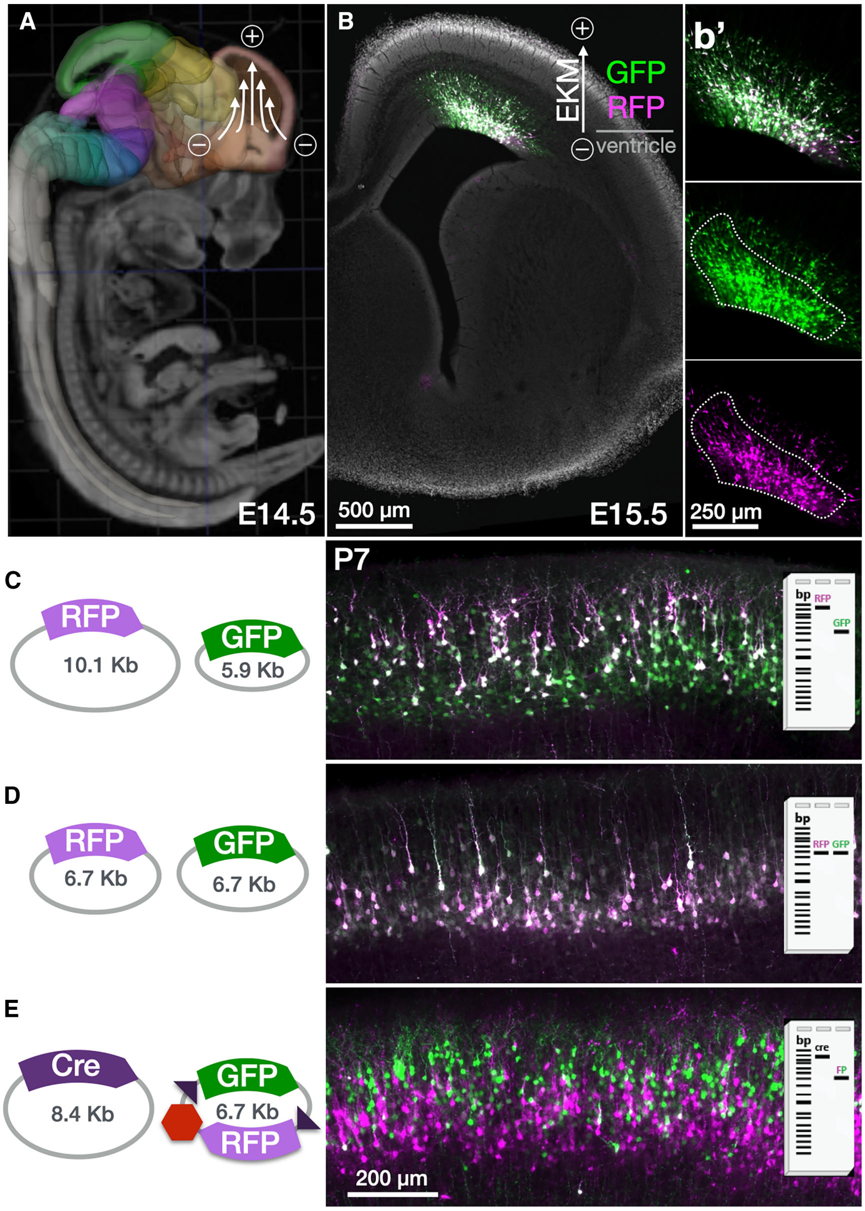

Figure 1. Segregation of plasmids by EKM within neural progenitor zones

(A) Schematic of tri-pole electrode (tritrode) *in utero* electroporation in the developing cerebral cortex (structure in orange, from Allen Developing Brain Atlas [ADBA]). Plasmid DNA injected in the lateral ventricle is targeted by a focused electric field into neural progenitors of the pallial neurogenic zone at the dorsal surface of the ventricle.

(B) Cells electroporated at E14.5 become labeled at E15.5 in the ventricular and intermediate zones within a wide range of depths. A dashed perimeter is indicated for reference to demonstrate higher penetration of labeling by the more mobile GFP plasmid compared to RFP (b′).

(C) Single E14.5 electroporation of an equimolar mixture of differentially sized plasmids (schematized as gel bands with different EKM) results in differential labeling of neurons at distinct layer depths in P7 cortex. Larger RFP plasmids labeled later-born superficial neurons (magenta), while smaller GFP plasmids labeled both superficial and earlier-born deeper layers (green).

(D) Size-matched GFP and RFP plasmids co-express in the same neurons without layer segregation.

(E) Titrated mixture of a large plasmid encoding Cre (8.4 kbp, 25 ng/μL) and a small (6.7 kbp, 2 μg/μL) bicistronic plasmid encoding conditional Cre-ON GFP (green) and Cre-OFF RFP (magenta), shows that neurons in a wider range of depths receive the small fluorophore plasmid (magenta and green cells), while only neurons closer to the ventricular surface and thus later-born upper-layer neurons receive the larger Cre plasmid to switch off RFP and switch on GFP (green cells). Specific plasmid sizes as indicated; black triangles indicate *loxP* sites, and the red hexagon indicates a transcriptional stop cassette. Full plasmid schemes are provided in Figure S1.

Scale bars: 500 μm in (A), 250 μm in (B), and 200 μm in (C), (D), and (E).

encodes Cre, such that older, deeper cells receive the fast plasmid and express RFP alone, while younger, shallower cells receive both fast and slow plasmids, suppressing RFP and expressing GFP alone (Figure 1E). With this conditional regime, we constructed plasmids with high expression of bright GFP and RFP variants, as well as optional modifications to facilitate membrane labeling (see STAR Methods and Figure S1).

Using bilateral tritrode electroporations, we titrated the amounts of plasmids to approach labeling parity in younger and older populations to achieve the highest FP expression, both of which were affected by Cre concentrations (Figure S3). A further internal gradation dependent on postmitotic cell age can be observed within each color, correlating broadly with fluorescence intensity. While intensity also varies stochastically from one cell to another based on plasmid copy number received, at the population level, intensity scales with age within each color population, such that brighter cells are generally younger/shallower than dimmer cells of the same color (Figures 1B, b′ and S3).

The layering effect was not region dependent, as the entirety of the electroporation field displayed differential labeling within layer II/III of all sections and throughout each *z* plane of the electroporation field with light sheet imaging (Figure 2). From our experience across hundreds of individual electroporations with a range of plasmids and a range of users, no plasmid pair has violated EKM rules (data not shown). In the data presented here, we quantify the position of stereo-tracked neurons from the pial surface and see a shifted range of expression along cortical layers, with RFP neurons on average positioned ~40 μm deeper than GFP neurons at P7, with plasmid pairs that are not Cre-dependent showing a similar range (Figures 2 and S3).

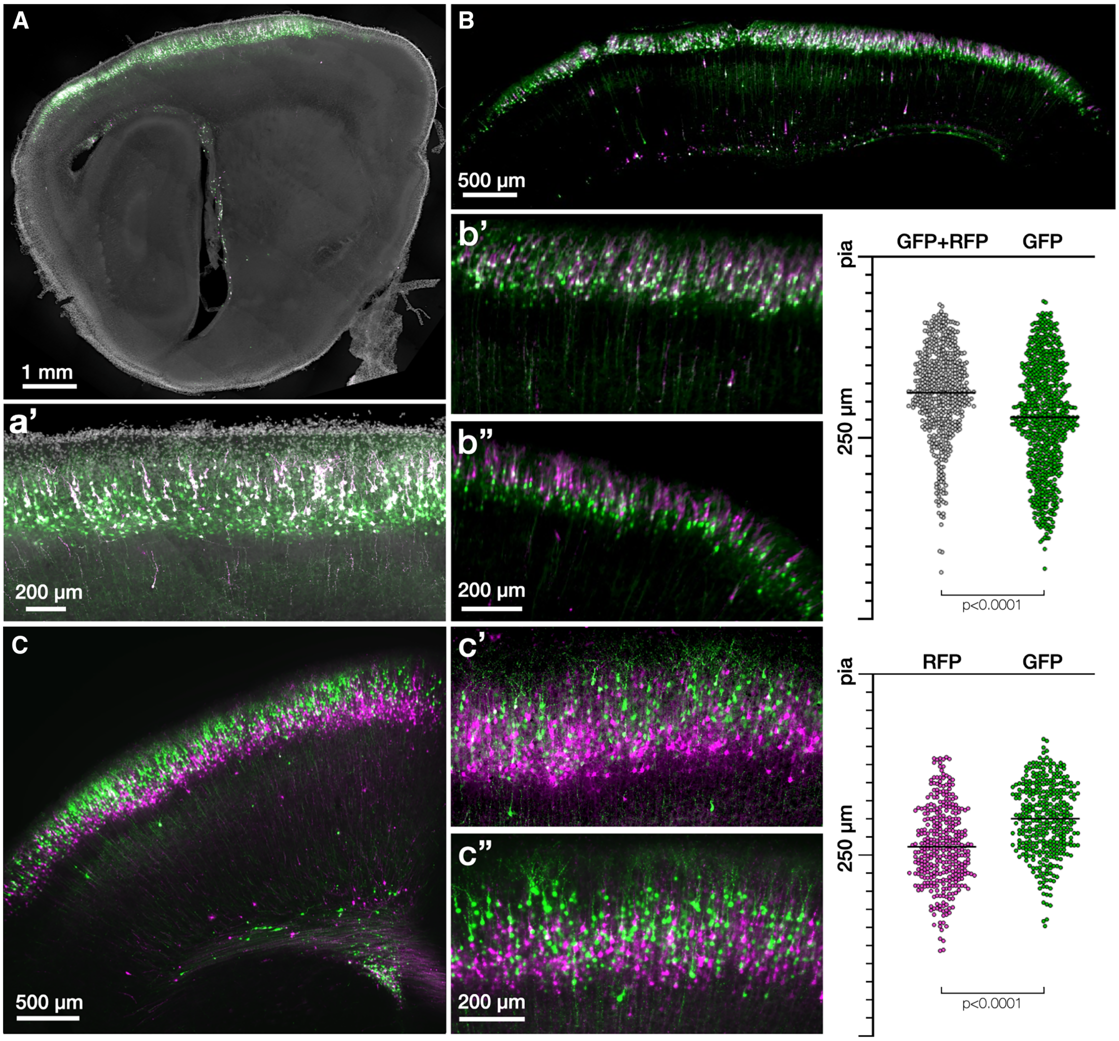

Figure 2. Stereo-tracked cells: Extent, modalities, variability, and quantification

(A) Epifluorescence imaging of the sagittal section displaying the extent of electroporation in the cerebral cortex using plasmids of Figure 1C (GFP, green; RFP, magenta; DAPI, white). Stereo-tracked cells demonstrate different labeling depths across cortical areas, as seen in the inset (a′), *N* = 3.

(B) Light sheet imaging of whole brain with plasmids as in (A), with magnified insets in (b′) and (b″). Swarm plots of cell depth quantification relative to the pial surface of differentially stereo-tracked cells from light sheet data. Each point is one cell, the black bar is the mean: RFP/GFP (gray) = 181.6 ± 60.2 μm; GFP (green) = 214.2 ± 73.9 μm.

(C) Epifluorescence imaging of coronal section displaying the complete electroporation field of Figure 1E. Additional replicates as shown in (c′) and (c″) with higher magnification. Quantification of layer position, as in (B): RFP (magenta) = 230.9 ± 52.6 μm; GFP (green) = 193.7 ± 47.7 μm, *N* = 3.

With these advances in understanding the distribution of electroporated cells based on plasmid EKM, progenitor zone stereo-tracking can achieve reproducible, staggered labeling of superficial vs. deeper layers in the cortex with a single electroporation, revealing migratory paths and topographic relationships along the cortical column. Stereo-tracking further offers the opportunity to investigate the progenitor zone patterning in brain regions with less-understood development.

Stereo-tracking in the cerebellum reveals topographic migration of Purkinje cells

We employed stereo-tracking to investigate the development of Purkinje cell migration in the cerebellum. Purkinje cells are born at E10–E12.5 in a compact progenitor zone above the 4th ventricle[16–19] (Figure S4). After neurogenesis, they migrate into the Purkinje plate,[11,20–22] undergo a period of clustering, and then disperse between P3–P10[7,23] into the foliating lobules and their characteristic pleated monolayer of the mature cerebellum.

We asked how the patterning of their progenitor zone contributes to Purkinje cell topographic patterning in the cerebellar cortex. To do so, we used stereo-tracking to segment nascent Purkinje cells, bisecting the fields of their neurogenic zone at E11.5. Using the conditional strategy of Figure 1E, EKM-discrepant plasmid mixture was injected into the 4th ventricle and electroporated to label deep cells with RFP and shallow cells with GFP (Figure 3). We observed striking topographic segregation of

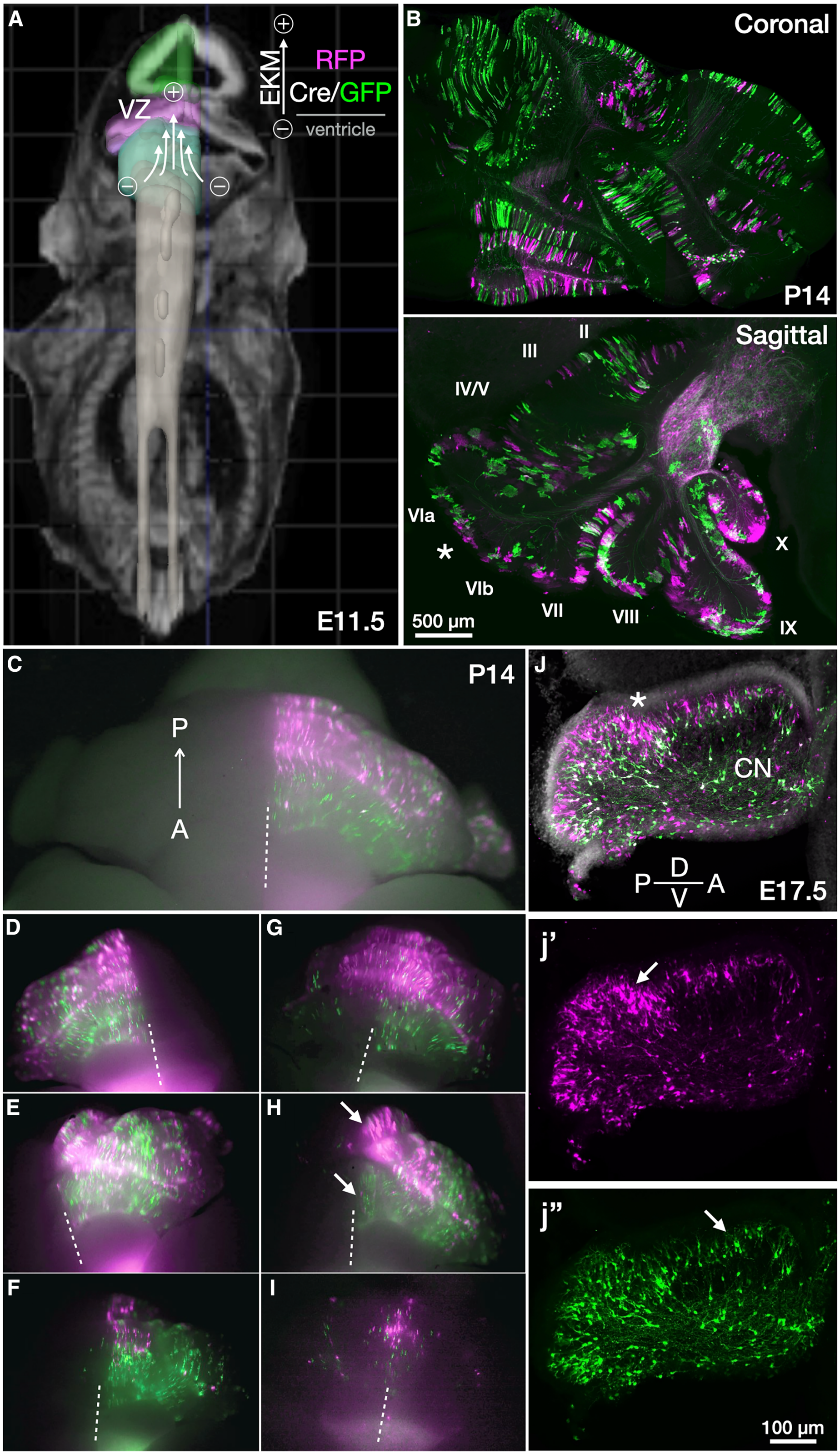

(legend on next page)

stereo-tracked Purkinje cells across entire lobules at P14. Both RFP and GFP are represented in the posterior cerebellum (Figure 3B, sagittal), with RFP cells highest in the vermis, while GFP dominated the anterior lobules (Figures 3C–3I). This pattern was reproducible across electroporations ($N = 10$).

The challenges of targeting the Purkinje cell progenitor zone, due to the early embryonic stage and central location,[9,24] resulted in varying levels of electroporation efficiency across individual electroporations, with the larger Cre plasmid being more variable due to its larger size. Through this intrinsic variability, we observed that in weaker electroporations, and/or when fertilization took place toward the later end of the overnight breeding window, the parasagittal stripe pattern previously reported to be dependent on Purkinje cell birthdate[4,5] becomes evident by stereo-tracking (Figure 3I). For example, the weak electroporation in Figure 3I leads to expression in only shallow-born cells at the ventricular surface, which corresponds to the parasagittal stripe pattern observed after E12.5 injection of birth-dated adenoviral vector expression.[5] Stronger electroporations delivered precisely at E11.5 label across parasagittal clusters and stripes as cerebellar compartmentalization matures (Figures 3C–3H).

The proximity of the embryo to the electrodes determines the strength of electroporation. When the electrical field is strong (e.g., Figure 3H), the posterior vermis contains the highest RFP expression, while the anterior regions are dimly GFP positive. This is due to the anterior region of the progenitor zone being intrinsically shallower (in terms of the number of cells stacked in the dorsal to ventral plane) than the posterior region (Figures 3J and S4; see also Vong et al.,[25] Khouri-Farah et al.,[26] and Tran-Anh et al.[27]), which means the majority of the progenitor field will receive low-EKM Cre and turn off RFP while turning on GFP. In the thicker posterior zone, its deep subfields will not receive low-EKM Cre, thus retaining many RFP-labeled cells. Correspondingly, in the neocortex, cells in deeper fields of the dorsal pallium get electroporated weakly, while those closer to the ventricular surface receive a higher copy number of FP plasmids and are therefore labeled brighter (Figure 1B, b′). This differential stereo-tracked labeling can be seen manifesting in the cerebellum at E17.5 (Figure 3J), like in early neocortex (Figure 1B).

The asterisks in Figures 3B and 3J highlight the same strongly electroporated posterior area at E17.5 and P14. Arrows in Figures 3H and 3J highlight bright cells near the border of the posterior and anterior lobules, which correspond to the posterior vermis at P14 (Figures 3C–3I). Therefore, individual Purkinje cells do not disperse randomly from the cluster stage into the Purkinje cell layer. Rather, Purkinje cells in the same progenitor zone field are embryonically committed to follow stereotyped trajectories leading them to specified topographic fields within the lobules of the adult cerebellar cortex (Figure 4).

A summary of stereo-tracked electroporation patterns in the cerebellum is schematized in Figure 4A. The shape of the Purkinje cell plate resembles a cornucopia, with the deepest fields of the posterior zone only receiving high-EKM RFP plasmid, leading to the high density of RFP stereo-tracked cells in the vermis. Lowering Cre concentration or electroporation efficiency shifts gradients to more RFP cells (Figure 3G), while elevating Cre concentrations leaves only the peak of the progenitor zone stereo-tracked with RFP, unreached by the large Cre plasmid (Figure 3F). Since the Purkinje cell plate is thinner in the anterior and lateral ventricular zone (VZ), the binary fluorophore effects become less pronounced outside the vermis.

Depending on the features of the progenitor zone within the electric field, stereo-tracking will reveal different patterns, as seen in the midbrain (Figure 4B). With size-matched plasmids and accurately titrated Cre (Figures 4C and S3), cells are equally labeled with RFP and GFP across the entirety of the cerebellum (Figure S5B). Cells in the anterior lobules generally develop later morphologically than posterior lobules,[22,28] as evidenced by progression of dendritic development (insets of Figure 4C), adding to the utility of this approach for directly comparing morphology and developmental profiles between Purkinje cell subpopulations.

Sparse GPI-anchored labeling revealed axon bubbles in Purkinje cell development

To enhance the labeling versatility of the stereo-tracking toolkit, we added lipid modifications to produce glycosylphosphatidylinositol (GPI)-anchored FP stereo-tracking constructs. The concentration of lipid-modified FPs along the perimeter of neurons

Figure 3. Stereo-tracking Purkinje cells in the developing cerebellum

(A) Tri-pole electrode (tritrode) electroporation schematic (ADBA) to target Purkinje cells in the VZ superior to the 4th ventricle at E11.5. Stereo-tracking plasmid pairs with GPI-anchored FPs (Figure S1D) label deeper cells with RFP-GPI (magenta) and shallower cells with Cre/GFP-GPI (green) as indicated in the EKM schematic.

(B) Coronal and sagittal sections at P14 produce complex patterns of stereo-tracked Purkinje cells in convoluted patterns that are difficult to appreciate in 2D sections.

(C) Epifluorescence stereoscope overview of whole uncleared cerebellum with stereo-tracked Purkinje cells on one hemisphere. The midline is indicated with a dashed line. Clear segregation of stereo-tracked cells can be seen along the anteroposterior axis (A→P). Strong electroporations include most lateral hemisphere portions and the flocculus.

(D–I) Further biological replicates of (C), with stereo-tracking in one or both (G) and (I) hemispheres. Strength of the electrical field and embryonic age in hours affect labeling outcomes; therefore, only ratiometric interpretations of stereo-tracked populations relative to each other should be made. The posterior vermis consistently shows the highest proportion of deep-labeled RFP cells and the highest exclusion of shallow-labeled Cre/GFP cells. Cre/GFP cells are predominant in anterior lobules, while the deep-labeled RFP cells (magenta) are predominant in posterior lobules. For size reference of stereoscope images in (C)–(I), see scale bar in equivalent 2D sample of P14 stereo-tracked cerebellum in (B).

(J) Sagittal section at E17.5 showing a distinct layering of differentially stereo-tracked migrating Purkinje cells of the posterior lobule, similar to that seen in the cerebral cortex. Asterisks in (B) and (J) indicate the separation point of posterior from anterior lobules. Arrows indicate majority RFP (j′) and GFP (j″) clusters at E17.5 and their corresponding positions at P14 (H).

Scale bars: 500 μm in (B) and 100 μm in (j″).

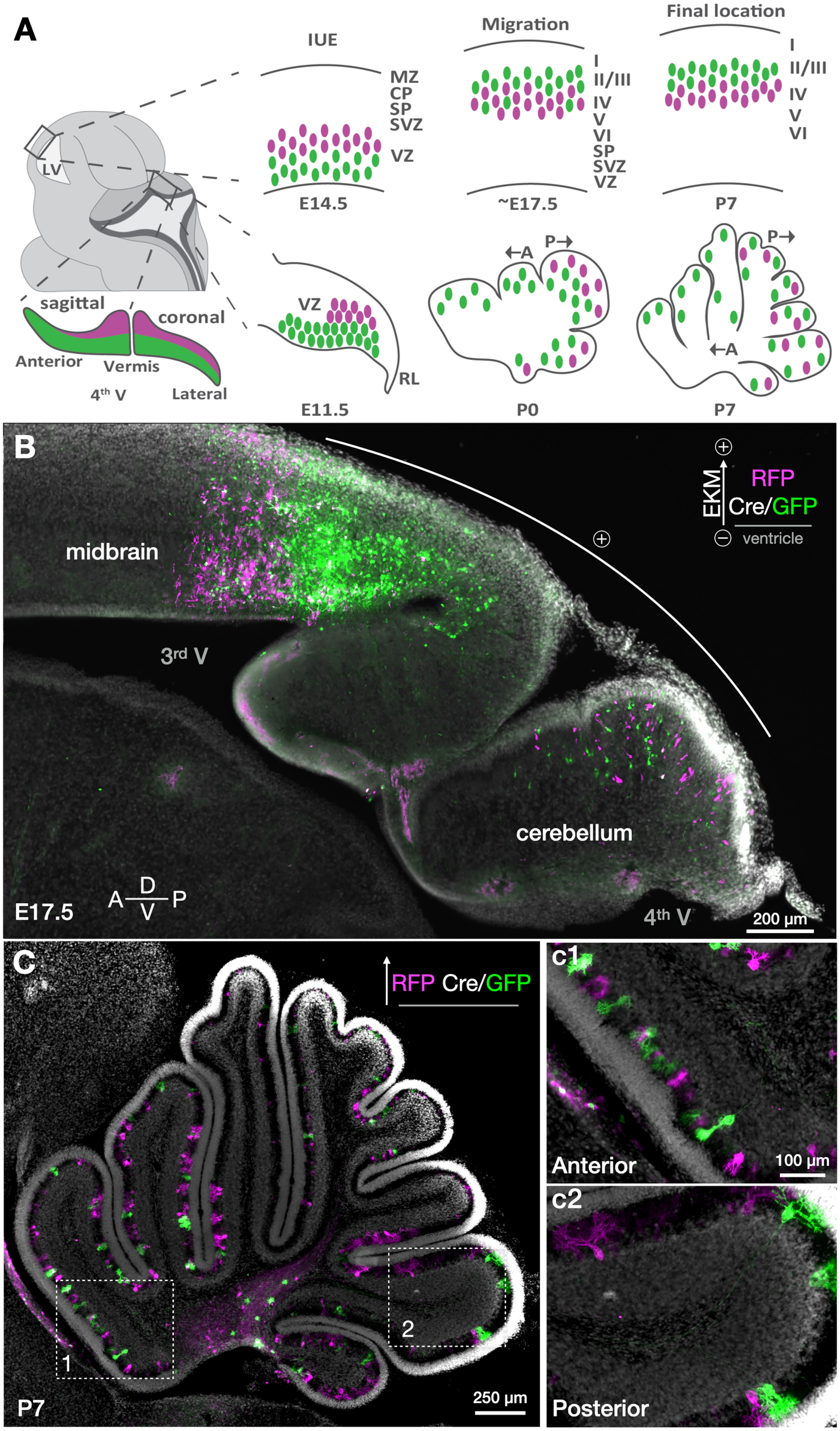

(legend on next page)

together with the sparse labeling achieved by *in utero* electroporation facilitates *in vivo* analyses of cellular morphologies, including axons and dendrites. Labeling with the GFP-GPI and RFP-GPI variants in the cerebellum (Figure S1D) revealed an unexpected subcellular structure emerging from developing Purkinje cell axons at the axon hillock and pinceau (Figures 5B–5D). These structures, which we term axon bubbles, were not present at P7 but appeared by P14, specifically at the interface of the Purkinje cell layer and the nascent granule cell layer across all areas of the cerebellum.

This is the phase of cerebellar development in which granule cell bodies migrate *en masse* through the Purkinje cell bodies to reposition from the superficial molecular layer to the nascent granule cell layer.[29] It is also the time when transient synapses of mossy fiber terminals onto Purkinje cell bodies and axon initial segments are eliminated to yield the mature circuitry of mossy fiber-to-granule cell innervation.[30] At P14, we see axon bubbles in roughly ∼20% of labeled Purkinje cells (Figure 5B), suggesting that these cellular protrusions appear transiently and may be involved in the dynamic cellular processes occurring at this developmental time point.

Cytosolic labeling has previously revealed swellings and torpedo structures in Purkinje cell axons.[31–33] Surprisingly, we did not observe labeling of axon bubbles with cytosolic FPs (Figure S5C), suggesting axon bubbles are distinct from axon swellings and torpedos, containing little if any accessible cytosol. Even more surprisingly, axon bubbles were not labeled by a myristoylated RFP (myr-RFP), which–like GPI–is known to label fine membranous processes of neurons *in vivo*,[34] albeit tethered on the inner, cytosolic-facing leaflet of cellular membranes (Figure 5A). These observations suggest that axon bubbles are either an artifact caused by FP-GPI overexpression or that the content within axon bubbles tightly excludes cytosolic components, possibly by an organelle or condensate, such that only GPI-anchored FPs on the outer surface of the plasma membrane can be accommodated (Figure 5D).

We turned to transmission electron microscopy (TEM) to determine whether axon bubbles were artifacts of electroporation and FP-GPI expression or were, in fact, previously unknown features of the developing cerebellum. Micrographs of outbred, non-electroporated, unlabeled CD1 mouse strain cerebella at P14 confirmed the presence of putative axon bubbles immediately adjacent to Purkinje cell axon initial segments (Figure 5E), consistent with those seen with FP-GPIs. These spherical electrolucent structures were specifically observed at the interface of the Purkinje cell layer and the nascent granule cell layer, as seen with electroporation, and not observed elsewhere within the cerebellar tissue. Importantly, these structures, which may be mistaken for the lumenal voids of blood vessels, are not surrounded by endothelial cells as is characteristic of blood vessels (Figure S6), though the cursory semblance may have contributed to them being previously unseen.

We quantified the morphometric features of the putative axon bubbles, including diameter and position, together with basic Purkinje cell measures of soma and nuclear diameter, in both electron micrographs and fluorescence microscopy (FM) images (Figure 5C). All measures showed tight correspondence in the two imaging modalities, with anticipated reduced sizes of soma, nucleus, and bubble diameter in TEM, attributed to fluorescence images being maximal projections of the entire structures, while electron micrographs sample random 50-nm cross-sections, which may not be the diameter maxima. The morphometric correspondence in the two very different imaging modalities confirmed that the electrolucent structures in electron micrographs indeed represent bona fide axon bubbles, and that axon bubbles are indeed features of the developing cerebellum, and not artifacts of electroporation or expression of FP-GPIs, as animals prepped for TEM were neither electroporated nor expressed any exogenous proteins.

These findings reveal a cellular structure, axon bubbles, that emerge in the second week of postnatal cerebellar development. Axon bubbles form 10–20 μm from the Purkinje cell soma, in other words, within one soma diameter's length. Axon bubbles average approximately 10 μm in diameter, approximately the size of Purkinje cell nuclei. Their interiors are particularly electrolucent and exclude cytosolic components and labels. Electrolucent bubbles are tightly surrounded by a single lipid membrane around most of their circumference. They do not contain recognizable axonal structures such as microtubules, neurofilaments, or other matrices, but may contain sporadic inclusions surrounded by membranes. This is in contrast to TEM images of axonal swellings[33] that have clear cytoplasmic structures, are surrounded by myelin, and form further along the axon proper, not at the axon initial segment. We observed torpedo/swelling-like structures in electroporations (Figure 5D arrowhead) in distinct positions and with distinct morphology from axon

Figure 4. Embryonic commitment of postnatal Purkinje cell topography in cerebellar cortex

(A) Model of Purkinje cell development based on stereo-tracking data. Cerebellar VZ gradients of stereo-tracked cells are schematized along anteroposterior and mediolateral axes. The anterior lobule has a shallower intermediate zone (see Figures 3J and S4) and becomes preferentially populated with shallow-labeled (green) migrating Purkinje cells. The posterior zone has a deeper intermediate zone with a higher proportion of deep-labeled cells (magenta). Purkinje cells do not migrate past earlier-born cells like neurons in the cerebral cortex; rather, they radiate outward in sequence with their position in the progenitor zone toward the outer cerebellar surfaces. Once the Purkinje cell monolayer forms (∼P7), most anterior cells derive from shallow VZ subfields (green), while the posterior zone comprises a mosaic of both deep- and shallow-labeled cells.

(B) Sagittal section of E17.5 midbrain and cerebellum, stereo-tracked at E11.5. Targeting cerebellar VZ in the 4th ventricle often leads to parallel targeting of the midbrain by the 3rd ventricle, with midbrain showing distinct patterns of stereo-tracking. Here, the anterior cerebellum was not electroporated efficiently. Firm placement of the electrode in line with the desired progenitor zone is critical for efficient electroporations.

(C) EKM-matched plasmids yield matched electroporation fields with dispersed labeling of RFP (magenta) and GFP (green). This enables sparse labeling of individual cells without biases of progenitor zone subfields. A natural developmental gradient can be seen at P7 where anterior lobules have Purkinje cells with more immature dendritic morphologies (dashed box 1 and inset c1) compared to the more developed arbors of the posterior cerebellum (dashed box 2 and inset c2). Some Purkinje cells can still be seen migrating along axon tracts, highlighting the protracted nature of cerebellar development.

Scale bars: 200 μm in (B), 250 μm in (C), and 100 μm in (c1).

CellPress
OPEN ACCESS

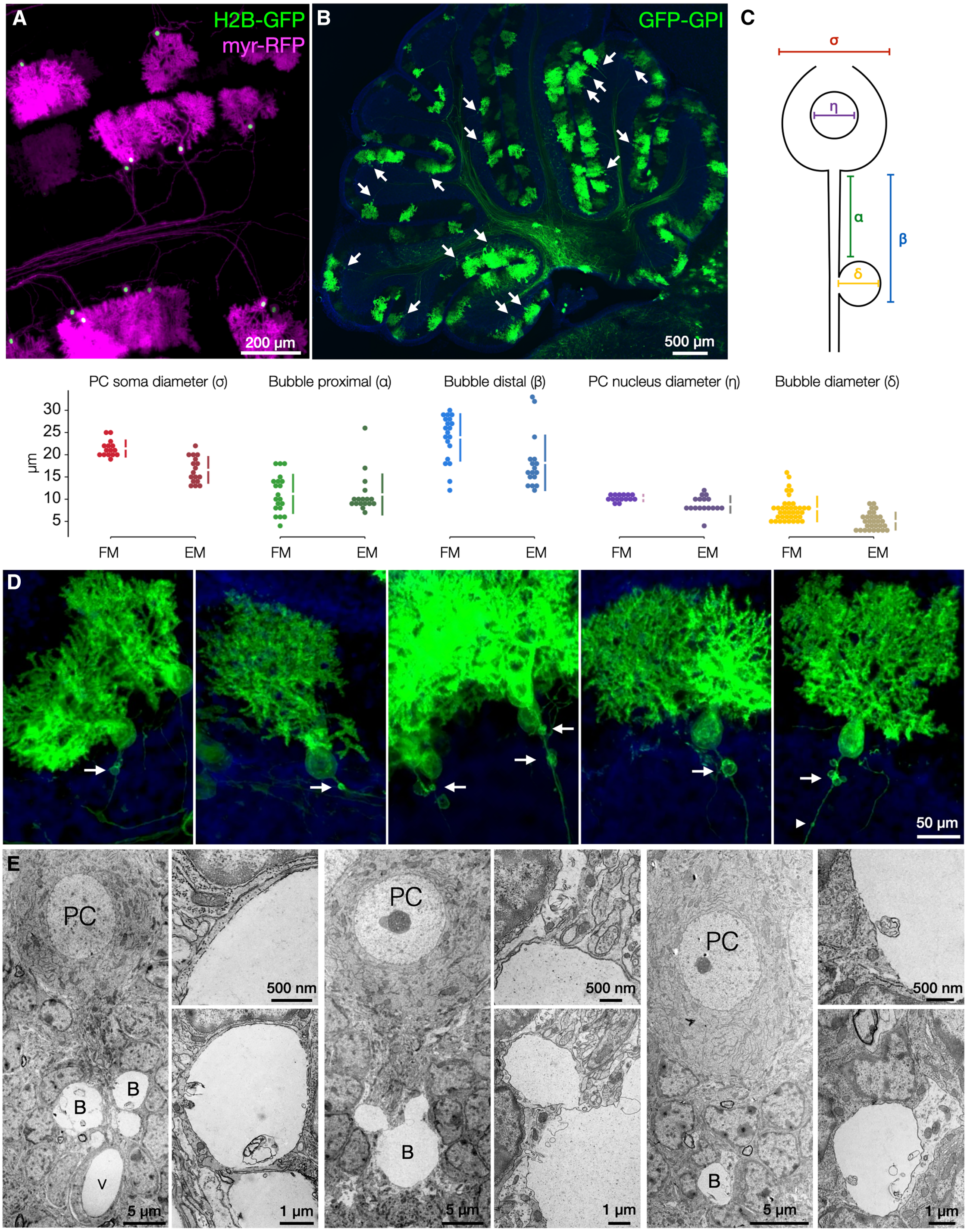

Figure 5. GPI-anchored fluorophores reveal Purkinje axon bubbles

(A) Electroporated Purkinje cells at P14 expressing membrane-localized myristoylated RFP (myr-RFP, magenta) and nuclear-localized Histone 2B fused to GFP (H2B-GFP, green). Inner leaflet myr-RFP outlines dendritic arbors and axons, but not axon bubbles.

(B) GPI-anchored GFP (GFP-GPI in green and DAPI in blue) illuminates fine membranous processes. Outer leaflet membrane labeling with GFP-GPI revealed previously unknown axon bubbles (arrows) at the axon hillocks of P14 Purkinje cells.

(C) Schema and swarm plots of Purkinje cell morphometric measurements as indicated in FM (D) and electron microscopy (EM) (E) datasets. Gapped lines in plots represent the mean (gap) and SD (lines). FM: $\sigma = 21.4 \pm 1.8$; $\eta = 10.3 \pm 0.7$*; $\alpha = 11.2 \pm 4.3$; $\beta = 23.9 \pm 5.2$; and $\delta = 7.8 \pm 2.8$ (*n* = 20; **n* = 15). EM: $\sigma = 16.6 \pm 2.9$; $\eta = 8.9 \pm 1.8$; $\alpha = 11.1 \pm 4.5$; $\beta = 18.2 \pm 6.2$; and $\delta = 5.1 \pm 1.9$ (*n* = 17).

(legend continued on next page)

bubbles, while swellings were also observed with cytoplasmic-facing FPs, unlike bubbles (Figure 5A). Overall, we observed axon bubbles in approximately 20% of electroporated Purkinje cells with FP-GPIs (*N* = 5 brains), while no axon bubbles were observed with myr-RFP (*N* = 3 brains) or cytosolic FPs (*N* = 4 brains), while a consistent presence was observed in all brains prepared for TEM experiments (*N* = 3). We hope the report of their discovery and the singular method of labeling by FP-GPIs will enable future studies to illuminate the prevalence, structure, and function of axon bubbles in brain development.

Optimizing stereo-tracking plasmids for 3D imaging by light sheet microscopy

The convolutions of the Purkinje cell layer along the cerebellar foliations make analyzing its topography particularly challenging through histological sectioning. To appreciate the topographic patterns of Purkinje cell migration, we used tissue clearing and light sheet microscopy[35] to document the positional relationships between stereo-tracked cells within the intact developing cerebellum in 3D.

We imaged a developmental time-course of the cleared, stereo-tracked cerebellum. To achieve sufficient processing throughput and favorable optical qualities, we optimized the labeling and clearing process[36] to maximally preserve endogenous fluorescence while enabling imaging deep into the cerebellar fissures (see STAR Methods). This enabled us to avoid antibody staining, thereby dramatically shortening processing times and lowering background. To better resolve cell morphology, including dendritic arbors and axon projections, we produced membrane-tethered versions of stereo-tracking plasmids using GPI-anchored FPs for membrane labeling (Figures 3B, 5B, and 6B). With these modifications and by using high quantum-yield variants of RFP and GFP (mScarlet-I[37] and mGreen Lantern,[38] respectively), we were able to rapidly clear brains while maintaining robust endogenous fluorescence.

With further modifications to the perfusion protocol and clearing with Cubic,[36] FP-GPIs tolerated clearing conditions well, with visualization of axon projections and dendritic arbors significantly improved over cytoplasmic labeling (Figure 6). Cold temperatures were critical for preserving plasmid supercoiling (Figure S2) and endogenous fluorescence during tissue processing. As such, plasmids were handled on ice, perfusion solutions were cold, and fixed brains were maintained chilled. To clear in CUBIC-L, 37°C is required, so this step was limited as much as possible to preserve endogenous fluorescence. Cubic-R shaking was limited to 2 days at room temperature to minimize impact on FPs. These workflow modifications enabled us to perform a developmental time-course of 3D maps of stereo-tracked Purkinje cells with their projections in the mouse cerebellum (Videos S1–S3).

Insights from stereo-tracking into cerebellar development

Purkinje cells are reported to be orchestrators of cerebellar circuit development.[39,40] Thus, understanding their migration patterns and developmental transformations informs many aspects of cerebellar development. We therefore followed the progression of generation-tracked Purkinje cells at three time points in the first 2 weeks of life with 3D cellular imaging of intact cerebella.

In utero electroporation allows for specific and sparse labeling of Purkinje cells by targeted electrical pulses to the progenitor zone during their brief window of birth. We used non-Cre cytoplasmic stereo-tracking plasmids (Figure 1C) across three postnatal developmental stages (P0, *N* = 2; P7, *N* = 1; and P14, *N* = 2). In this regimen, the low-EKM RFP plasmid was electroporated less efficiently overall, with no significant labeling of the anterior cerebellum. The high-EKM GFP plasmid efficiently electroporated both anterior and posterior areas, as well as many more neurons (17.2-fold more GFP than RFP cells). When using Cre-dependent membrane-bound stereo-tracking plasmids (Figure 6B), low-EKM Cre/GFP transfects cells at the ventricular surface, and since the high-EKM RFP/GFP plasmid dictates fluorophore expression, small amounts of Cre recombinase are able to change the majority of the cells to GFP. This is in contrast to large fluorophore plasmids that are often too dim to image with a light sheet due to low copy numbers. Changing Cre concentrations determines the ratio of GFP:RFP labeling.

At P0 (Figure 6A), nascent postmitotic Purkinje cells radiate away from their progenitor zone, and deeper-labeled Purkinje cells are farther along their migratory route toward the Purkinje plate. While later-born shallow-labeled pyramidal cells of the neocortex push past earlier-born, deep-labeled pyramidal cells to populate different layers of the cortical column, deep-labeled Purkinje cells join shallow-labeled cells in the same monolayer. Anterior lobules are overwhelmingly shallow-labeled due to the narrower depth-of-field of the anterior progenitor zone at E11.5. Similar areas of the VZ must also accommodate neurogenesis of cerebellar nucleus interneurons from E10–E12 in the area expressing Gsx1.[41] The anterior and lateral portions of the developing cerebellum have less overall Purkinje cell numbers and multicellular layering within clusters in the anteroposterior plane[27] (Figure S4) and Purkinje cells born on E10.5 are a small subset in the anterior zone, biasing them toward a GFP label.[42] By P7, cells have largely reached their position within the Purkinje cell layer. Electrode placement determines the extent of the electroporation field, with successful placement at the midline leading to robust labeling of the vermis and labeling of sagittal stripes (Figure 6 and P7 Video S2).

Together, cerebellum stereo-tracking reveals that Purkinje cells remain in the general quadrants in which they were born in the VZ, similar to previous reports.[4,29] Cells born along the

(D) Individual GFP-GPI-labeled Purkinje cells from (B), with arrows indicating axon bubbles. An arrowhead marks a torpedo-like structure, distinct from axon bubbles.

(E) Transmission electron micrographs of unmanipulated P14 mouse cerebellum showing electrolucent axon bubbles (B) at a similar distance from the Purkinje cell body (PC), corresponding to the location seen in electroporated cells near the axon hillock. Distinct blood vessels are indicated (V), surrounded by vascular endothelium (see also Figure S6). Insets show higher magnifications of each image, focusing on axon bubble membranes.

Scale bars: 200 μm in (A), 500 μm in (B), 50 μm in (D), and 5 μm, 500 nm, and 1 μm, respectively, in (E).

CellPress
OPEN ACCESS

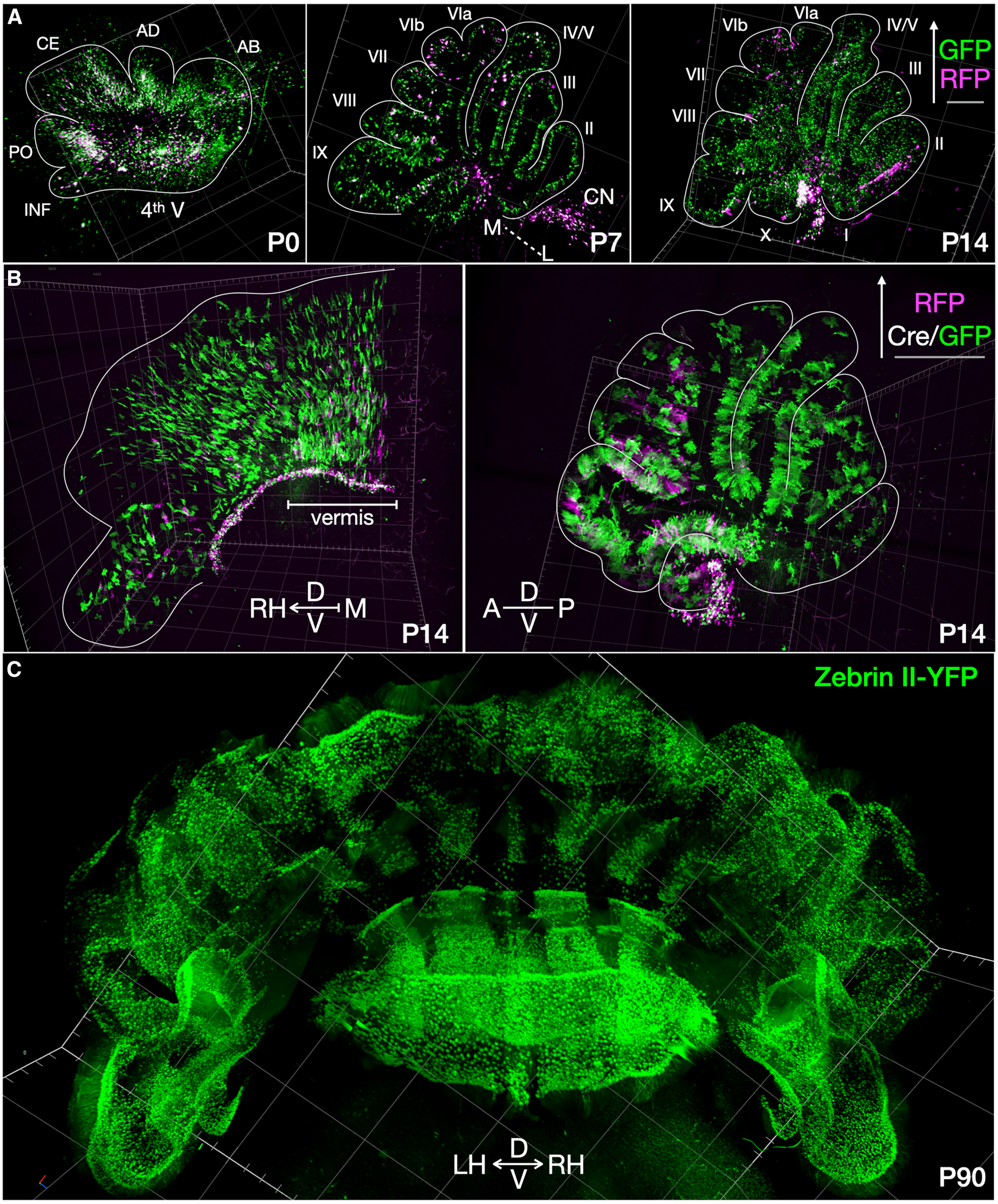

Figure 6. Light sheet imaging of whole cerebella after stereo-tracking across development

(A) 3D rendered projections of light sheet imaging of whole cerebella after stereo-tracking with cytosolic deep-labeled GFP (green) and shallow-labeled RFP (magenta) plasmids (Figure S1A) at three developmental time points as indicated. Posterior cerebellum consistently displays higher electroporation efficiency and a higher proportion of deep-labeled GFP cells. $N = 2$ P0, $N = 1$ P7, and $N = 2$ P14 brains were rendered in 3D to identify individual stereo-tracked Purkinje cells (see Video S2). There were over 15× more GFP cells than RFP cells in this labeling cohort.

(B) Light sheet 3D rendering (Video S1) with coronal (left) and sagittal (right) projection of stereo-tracked P14 cerebellum with deep-labeled RFP-GPI and shallow-labeled Cre/GFP-GPI plasmid pairs (Figure S1D). Sagittal view superimposes the entire lateral extent of the vermis and individual lobules across planes.

(C) Coronal projection of light sheet 3D rendering (Video S3) of adult cerebellum from a mouse strain expressing YFP fused to aldolase C (zebrin II), processed and imaged with an optimized clearing method, reveals contiguous and convoluted stripe topography. 3D videos better capture the topography of labeled Purkinje cells.

AB, anterobasal lobe; AD, anterodorsal lobe; CE = central lobe; PO, posterior lobe; and INF, inferior lobe. Each box tick mark is 200 μm in *XYZ* reference.

midline migrate outward to occupy the vermis (P7 Video S2), cells born more laterally (P14 Video S2) occupy the lateral hemispheres. Anterior-born cells reside in the mature anterior lobules, and posterior-born cells reside in the mature posterior lobules. These different quadrants show distinctly different rates of Purkinje cell maturation based on morphology within specific lobules (e.g., Figure 4C).

The subtypes of Purkinje cells, as defined by molecular markers, in these birth locations, remain unclear. However, combining our developmentally informed, molecularly agnostic labeling with specific markers presents an interesting future research direction to track how clusters transform into distinct parasagittal stripes. Since the different stripe markers far outnumber the clusters from which they emerge,[7] it is possible that deep- and shallow-labeled cells within one cluster, bisected by stereo-tracking, will have differential cascades of gene expression based on the progenitor zone field that commits their differentiation, manifesting in the observed mature stripe patterning. It is thought that compartmentalization begins as early as E10, with the cerebellar stripe arrays developing from the early dorsal to ventral layering in the VZ.[8] Thus, it will be useful to see where electroporated cells overlap with stripe markers using the sparse labeling strategy to more readily track individual cell transformations to their mature locations.

With our improved methods for preserving endogenous fluorescence, we tested whether transgenic lines could similarly be imaged with light sheet for tracking complicated stripe topography, such as that of aldolase C (zebrin II). P90 aldoc-venus mice[43] highlight the utility of whole cerebellar 3D imaging for examining circuit architecture (Figure 6C).

Purkinje cell patterning based on parasagittal stripe markers is typically represented in 2D by multiple sections. However, observing whole populations in their native circuit architecture allows for examining the continuity of parasagittal stripes across lobules, which was previously unknown for aldolase C, the most widely studied stripe marker.[44] For example, stripes in the cerebellar hemispheres appear less defined in sections than in the vermis; however, "ribbons" of aldolase C-positive cells connecting across and within lobules can be observed in 3D, which raises the question of how signaling across these Purkinje cell subtypes may coordinate their behaviors across the topography of the cerebellar cortex. Building accurate models of these topographical features will be important for generating hypotheses on the physiological function.

DISCUSSION

Here, we describe a bioelectric interaction of plasmids injected embryonically and targeted at neural progenitors by the pulsed application of an electric field. Delipidated brains have properties similar to an electrolyte gel,[45] and the differential labeling we see is consistent with an electrogenic phenomenon similar to DNA gel electrophoresis. Small, supercoiled plasmids penetrate deeper into the progenitor zone and thus target earlier-born cells of the neocortex on their way to becoming migrating postmitotic neurons. We took advantage of this phenomenon to develop stereo-tracking, a differential labeling strategy that narrowly distinguishes between cells at different depths within the volume of VZ progenitor subfields, and applied this strategy to investigate how progenitor zone location impacts cerebellar development.

Overall, EKM-mismatched mosaic plasmids can label shallow cells at the ventricular surface with the slower plasmid and deeper cells beyond that with the faster plasmid in both cerebral and cerebellar progenitor zones. However, while the progenitor zone depth of cortical cells is uniform, the cerebellum has a progenitor zone with a more complex 3D landscape in terms of the number of Purkinje cells born across days between the anterior and posterior regions. The embryonic spatial origins commit Purkinje cells already from E11.5 to the complex topographic patterns that they will assume in the adult cerebellar cortex.

Studies of Purkinje cell migration and rearrangement of clusters heavily rely on known markers at various stages.[3,4] Stereo-tracking allows the developmentally-informed tracking of Purkinje cells without regard to molecular subtypes, so that overall topography based on progenitor zone subfields can be observed. This approach is not reliant on marker staining or transgenic lines and has been a barrier to studying the development of Purkinje cells. Molecular markers are not stably expressed, making it difficult to track Purkinje cells from the immature cluster stage to locations within the cortex, especially in the significant transitions around P6[4].

Because Purkinje cells born on specific days lead to adult sagittal stripe patterning after the cluster stage,[5] the developmental migration patterns of Purkinje cells appear to be more complicated than other structures. We show with stereo-tracking that Purkinje cells follow a similar overall pattern of migration as in neocortex at early stages (E17.5-P3), in that migration of cells proceeds directly from the place of origin to the expanding layers above (Figure 4A). Cells deeper in the progenitor zone lead the way in pushing the superficial regions of the cerebellar cortex outward, and cells labeled at the shallow subfields of the VZ follow closely behind. However, unlike the inside-out layering of neocortex, where stereo-tracked cells populate distinct layers, later-born Purkinje cells populate distinct topographical fields within their monolayer in the mature cerebellar cortex.

Purkinje cell clusters have been reconstructed from 2D images into 3D representations[4] in an attempt to unravel the complex transition to adult stages. However, directly observing structures in 3D without sectioning is an advantage of light sheet microscopy and enables appreciation for the continuity of structures in the highly foliated cerebellum. Additionally, the flexibility of fluorophores afforded by plasmid-based labeling supports the identification of different cellular features, as demonstrated by our unexpected discovery of Purkinje cell axon bubbles.

Limitations of the study

The technical innovation of stereo-tracking offers a pulse-chase developmentally-informed labeling strategy that can be combined with other *in utero* electroporation technologies, including genome editing,[46,47] for studying brain development. It additionally highlights the importance of plasmid handling and size matching when co-electroporation of plasmid mixtures is desired to avoid unintended artifacts of labeling. Further technical improvements for labeling include the use of tritrode electroporation for higher efficiency and better targeting of the

Purkinje cell progenitor zone, supercoiled plasmids of small overall size to increase labeling efficiency, and cold-processing brain tissue to preserve endogenous fluorescence for light sheet imaging in 3D.

Labeling at early embryonic stages can have low survival rates, and whether electroporation and expression of plasmids alter the normal development of neurons is unclear. Contrary to what was previously assumed, plasmids not only electroporate cells directly on the ventricular surface but also cells somewhat deeper into the progenitor zone. Therefore, cells not born on the same day as the electroporation will also be electroporated, which can limit developmental interpretations if this effect is unintended. Particularly in the cerebellum, where patterning of embryonic clusters and mature stripes along multiple planes is informative, electroporation ignores these molecular and anatomical boundaries by pulsing across a certain volume of the VZ and targeting cells at different stages within the 3 days of Purkinje cell birth.

Carefully selecting EKM properties of plasmids and electric field orientation can help further identify how distinct subfields and depth of cells labeled at the progenitor zone translate into mature patterns of circuitry, both in the neocortex and the cerebellum. The basic parameters described can be tuned for a specific effect. For example, changing Cre concentrations can fine-tune labeling ratios, changing plasmid size can impact the penetration depth of cells electroporated, and plasmids can be designed with multiple genetic elements that give experimental flexibility. Pairing Cre-dependent lines with Cre plasmids provides additional important avenues of experimentation.

We document the presence of axon bubbles in stereotyped positions of P14 Purkinje axon initial segments. However, we have identified neither their function nor their mysterious internal content. Cryoelectron microscopy (EM) tomography would help better preserve and interpret the membranes of axon bubbles. Serial section reconstitution of TEM Purkinje cells would help determine the precise points of contact along the axon. While we speculate that axon bubbles are a transient feature of developing Purkinje cells due to their tight developmental onset and scattered presence, we have not determined whether they disappear in the mature cerebellum. Future studies will be useful to determine their matrix, function, and defined developmental window, as well as in targeted investigations on whether homologous structures may exist in other neuron types in development or in other animal species or mouse strains.

RESOURCE AVAILABILITY

Lead contact

Further information and requests for resources and reagents should be directed to and will be fulfilled by the lead contact, Alexandros Poulopoulos (apoulopoulos@som.umaryland.edu).

Materials availability

Plasmids used for stereo-tracking are available upon request through the lead contact.

Data and code availability

- Raw imaging and quantification data supporting the figures are available upon request.
- This paper does not report original code.
- Any additional information required to reanalyze the data reported in this paper is available from the lead contact upon request.

ACKNOWLEDGMENTS

We are grateful to Bekir Altas, Colin Robertson (Poulopoulos Lab, Baltimore), and Andy Cole (Reese lab, NIH) for very useful discussions and to Joseph Mauban and Thomas Blanpied for the technical and instrumentation support at the University of Maryland School of Medicine Center for Innovative Biomedical Resources through the Confocal Imaging Facility and grant S10OD030221 (Zeiss Light sheet). C.B. is exceptionally grateful to Dr. Greg Elmer for his support and mentorship throughout the project under the T32 fellowship.

This work was supported by the National Institutes of Health (USA) through the High-Risk, High-Reward Research Program of the National Institutes of Health Common Fund under award no. DP2MH122398 (A.P.) and the Autism Research Institute through grant nos. 30030461 (C.B.) and R01NS127435 (R.V.S.). C.B. was supported by the Schizophrenia & Psychosis-Related Disorders T32 training grant of the Maryland Psychiatric Research Center under award no. T32MH067533 and the Cancer Biology T32 Training Program at the University of Maryland School of Medicine funded by the National Cancer Institute under award no. T32CA154274. The project was supported in part by the University of Maryland Greenebaum Comprehensive Cancer Center support grant nos. P30CA134274 and S10OD030221 through core use, and the IDDRC grant no. P50HD103555 from the Eunice Kennedy Shriver National Institute of Child Health & Human Development, Neuropathology Core, and NRI TEM Core.

AUTHOR CONTRIBUTIONS

Conceptualization: C.B., G.W.C., and A.P.; methodology: C.B.; validation: G.W.C. and A.J.R.; formal analysis: C.B. and A.P.; investigation: C.B., S.G. D., and L.R.D.; resources: R.N.A.O.-M., I.S., R.V.S., and A.P.; writing – original draft: C.B.; writing – review and editing: C.B., G.W.C., A.J.R., S.G.D., L.R.D., R.N.A.O.-M., B.H.C., I.S., G.J.B., R.V.S., and A.P.; visualization: C.B., L.R.D., and A.P.; supervision: R.V.S. and A.P.

DECLARATION OF INTERESTS

The authors declare no competing interests.

STAR★METHODS

Detailed methods are provided in the online version of this paper and include the following:

- KEY RESOURCES TABLE
- EXPERIMENTAL MODEL AND STUDY PARTICIPANT DETAILS
- METHOD DETAILS
  - Plasmids
  - *In utero* electroporation
  - Titrating cre in EKM plasmid mixtures
  - Perfusion and tissue processing
  - Clearing and light sheet imaging
  - Transmission electron microscopy
- QUANTIFICATION AND STATISTICAL ANALYSIS

SUPPLEMENTAL INFORMATION

Supplemental information can be found online at https://doi.org/10.1016/j.crmeth.2025.101143.

Received: August 22, 2024
Revised: April 22, 2025
Accepted: July 30, 2025
Published: August 26, 2025

REFERENCES

1. Kittock, C.M., and Pilaz, L.J. (2023). Advances in in utero electroporation. Dev. Neurobiol. *83*, 73–90. https://doi.org/10.1002/dneu.22910.
2. Ma, J., Shen, Z., Yu, Y.-C., and Shi, S.-H. (2018). Neural lineage tracing in the mammalian brain. Curr. Opin. Neurobiol. *50*, 7–16. https://doi.org/10.1016/j.conb.2017.10.013.
3. Wizeman, J.W., Guo, Q., Wilion, E.M., and Li, J.Y. (2019). Specification of diverse cell types during early neurogenesis of the mouse cerebellum. eLife *8*, e42388–24. https://doi.org/10.7554/eLife.42388.
4. Fujita, H., Morita, N., Furuichi, T., and Sugihara, I. (2012). Clustered fine compartmentalization of the mouse embryonic cerebellar cortex and its rearrangement into the postnatal striped configuration. J. Neurosci. *32*, 15688–15703. https://doi.org/10.1523/JNEUROSCI.1710-12.2012.
5. Hashimoto, M., and Mikoshiba, K. (2003). Mediolateral Compartmentalization of the Cerebellum Is Determined on the "Birth Date" of Purkinje Cells. J. Neurosci. *23*, 11342–11351. https://doi.org/10.1523/jneurosci.23-36-11342.2003.
6. Beckinghausen, J., and Sillitoe, R.V. (2019). Insights into cerebellar development and connectivity. Neurosci. Lett. *688*, 2–13. https://doi.org/10.1016/j.neulet.2018.05.013.
7. Dastjerdi, F.V., Consalez, G.G., and Hawkes, R. (2012). Pattern formation during development of the embryonic cerebellum. Front. Neuroanat. *6*, 10. https://doi.org/10.3389/fnana.2012.00010.
8. Leto, K., Arancillo, M., Becker, E.B.E., Buffo, A., Chiang, C., Ding, B., Dobyns, W.B., Dusart, I., Haldipur, P., Hatten, M.E., et al. (2016). Consensus Paper: Cerebellar Development. Cerebellum *15*, 789–828. https://doi.org/10.1007/s12311-015-0724-2.
9. Dal Maschio, M., Ghezzi, D., Bony, G., Alabastri, A., Deidda, G., Brondi, M., Sato, S.S., Zaccaria, R.P., Di Fabrizio, E., Ratto, G.M., and Cancedda, L. (2012). High-performance and site-directed in utero electroporation by a triple-electrode probe. Nat. Commun. *3*, 960. https://doi.org/10.1038/ncomms1961.
10. Maniatis, T., Jeffrey, A., and van deSande, H. (1975). Chain length determination of small double- and single-stranded DNA molecules by polyacrylamide gel electrophoresis. Biochemistry *14*, 3787–3794. https://doi.org/10.1021/bi00688a010.
11. Rakic, P., and Sidman, R.L. (1970). Histogenesis of cortical layers in human cerebellum, particularly the lamina dissecans. J. Comp. Neurol. *139*, 473–500. https://doi.org/10.1002/cne.901390407.
12. Cossart, R., and Garel, S. (2022). Step by step: cells with multiple functions in cortical circuit assembly. Nat. Rev. Neurosci. *23*, 395–410. https://doi.org/10.1038/s41583-022-00585-6.
13. Taylor, R.J., Carrington, J., Gerlach, L.R., Taylor, K.L., Richters, K.E., and Dent, E.W. (2020). Double UP: A Dual Color, Internally Controlled Platform for in utero Knockdown or Overexpression. Front. Mol. Neurosci. *13*, 82. https://doi.org/10.3389/fnmol.2020.00082.
14. Greig, L.C., Woodworth, M.B., Poulopoulos, A., Lim, S., and Macklis, J.D. (2023). BEAM: a combinatorial recombinase toolbox for binary gene expression and mosaic analysis. Preprint at bioRxiv. https://doi.org/10.1101/2023.02.16.528875.
15. Poulopoulos, A., Davis, P., Brandenburg, C., Itoh, Y., Galazo, M.J., Greig, L.C., Romanowski, A.J., Budnik, B., and Macklis, J.D. (2024). Symmetry in levels of axon-axon homophilic adhesion establishes topography in the corpus callosum and development of connectivity between brain hemispheres. Preprint at bioRxiv. https://doi.org/10.1101/2024.03.28.587108.
16. Mathis, L., Bonnerot, C., Puelles, L., and Nicolas, J.-F. (1997). Retrospective clonal analysis of the cerebellum using genetic laacZ/lacZ mouse mosaics. Development *124*, 4089–4104. https://doi.org/10.1242/dev.124.20.4089.
17. Miale, I.L., and Sidman, R.L. (1961). An autoradiographic analysis of histogenesis in the mouse cerebellum. Exp. Neurol. *4*, 277–296. https://doi.org/10.1016/0014-4886(61)90055-3.
18. Pierce, E.T. (1975). Histogenesis of the deep cerebellar nuclei in the mouse: an autoradiographic study. Brain Res. *95*, 503–518. https://doi.org/10.1016/0006-8993(75)90124-9.
19. Altman, J., and Bayer, S.A. (1978). Prenatal development of the cerebellar system in the rat. I. Cytogenesis and histogenesis of the deep nuclei and the cortex of the cerebellum. J. Comp. Neurol. *179*, 23–48. https://doi.org/10.1002/cne.901790104.
20. Goffinet, A.M. (1983). The embryonic development of the cerebellum in normal and reeler mutant mice. Anat. Embryol. *168*, 73–86. https://doi.org/10.1007/BF00305400.
21. Gu, C., and Gu, Y. (2011). Clustering and activity tuning of Kv1 channels in myelinated hippocampal axons. J. Biol. Chem. *286*, 25835–25847. https://doi.org/10.1074/jbc.M111.219113.
22. Yuasa, S., Kawamura, K., Ono, K., Yamakuni, T., and Takahashi, Y. (1991). Development and migration of Purkinje cells in the mouse cerebellar primordium. Anat. Embryol. *184*, 195–212. https://doi.org/10.1007/BF01673256.
23. Rahimi-Balaei, M., Bergen, H., Kong, J., and Marzban, H. (2018). Neuronal migration during development of the cerebellum. Front. Cell. Neurosci. *12*, 484. https://doi.org/10.3389/fncel.2018.00484.
24. Szczurkowska, J., Cwetsch, A.W., Dal Maschio, M., Ghezzi, D., Ratto, G. M., and Cancedda, L. (2016). Targeted in vivo genetic manipulation of the mouse or rat brain by in utero electroporation with a triple-electrode probe. Nat. Protoc. *11*, 399–412. https://doi.org/10.1038/nprot.2016.014.
25. Vong, K.I., Leung, C.K.Y., Behringer, R.R., and Kwan, K.M. (2015). Sox9 is critical for suppression of neurogenesis but not initiation of gliogenesis in the cerebellum. Mol. Brain *8*, 25. https://doi.org/10.1186/s13041-015-0115-0.
26. Khouri-Farah, N., Guo, Q., Morgan, K., Shin, J., and Li, J.Y.H. (2022). Integrated single-cell transcriptomic and epigenetic study of cell state transition and lineage commitment in embryonic mouse cerebellum. Sci. Adv. *8*, eabl9156–18. https://doi.org/10.1126/sciadv.abl9156.
27. Tran-Anh, K., Zhang, J., Nguyen-Minh, V.T., Fujita, H., Hirata, T., and Sugihara, I. (2020). Common Origin of the Cerebellar Dual Somatotopic Areas Revealed by Tracking Embryonic Purkinje Cell Clusters with Birthdate Tagging. eNeuro *7*, ENEURO.0251. https://doi.org/10.1523/ENEURO.0251-20.2020.
28. Beekhof, G.C., Osório, C., White, J.J., van Zoomeren, S., van der Stok, H., Xiong, B., Nettersheim, I.H., Mak, W.A., Runge, M., Fiocchi, F.R., et al. (2021). Differential spatiotemporal development of Purkinje cell populations and cerebellum-dependent sensorimotor behaviors. eLife *10*, e63668. https://doi.org/10.7554/eLife.63668.
29. Sillitoe, R.V., Gopal, N., and Joyner, A.L. (2009). Embryonic origins of ZebrinII parasagittal stripes and establishment of topographic Purkinje cell projections. Neuroscience *162*, 574–588. https://doi.org/10.1016/j.neuroscience.2008.12.025.
30. Kalinovsky, A., Boukhtouche, F., Blazeski, R., Bornmann, C., Suzuki, N., Mason, C.A., and Scheiffele, P. (2011). Development of axon-target specificity of ponto-cerebellar afferents. PLoS Biol. *9*, e1001013. https://doi.org/10.1371/journal.pbio.1001013.
31. Cajal, S.R.y., DeFelipe, J., and Jones, E.G. (1991). In Cajal's Degeneration and Regeneration of the Nervous System J. DeFelipe, E.G. Jones, ed. (Oxford University Press). https://doi.org/10.1093/acprof:oso/9780195065169.001.0001.
32. Bouman, L. (1918). Die Histopathologie Der Psychosen (Springer).
33. Lang-Ouellette, D., Gruver, K.M., Smith-Dijak, A., Blot, F.G.C., Stewart, C. A., de Vanssay de Blavous, P., Li, C.H., Van Eitrem, C., Rosen, C., Faust, P. L., et al. (2021). Purkinje cell axonal swellings enhance action potential fidelity and cerebellar function. Nat. Commun. *12*, 4129. https://doi.org/10.1038/s41467-021-24390-4.
34. Poulopoulos, A., Murphy, A.J., Ozkan, A., Davis, P., Hatch, J., Kirchner, R., and Macklis, J.D. (2019). Subcellular transcriptomes and proteomes of developing axon projections in the cerebral cortex. Nature *565*, 356–360. https://doi.org/10.1038/s41586-018-0847-y.

35. Hillman, E.M.C., Voleti, V., Li, W., and Yu, H. (2019). Light-Sheet Microscopy in Neuroscience. Annu. Rev. Neurosci. *42*, 295–313. https://doi.org/10.1146/annurev-neuro-070918-050357.

36. Matsumoto, K., Mitani, T.T., Horiguchi, S.A., Kaneshiro, J., Murakami, T. C., Mano, T., Fujishima, H., Konno, A., Watanabe, T.M., Hirai, H., and Ueda, H.R. (2019). Advanced CUBIC tissue clearing for whole-organ cell profiling. Nat. Protoc. *14*, 3506–3537. https://doi.org/10.1038/s41596-019-0240-9.

37. Bindels, D.S., Haarbosch, L., van Weeren, L., Postma, M., Wiese, K.E., Mastop, M., Aumonier, S., Gotthard, G., Royant, A., Hink, M.A., and Gadella, T.W.J., Jr. (2017). mScarlet: a bright monomeric red fluorescent protein for cellular imaging. Nat. Methods *14*, 53–56. https://doi.org/10.1038/nmeth.4074.

38. Campbell, B.C., Nabel, E.M., Murdock, M.H., Lao-Peregrin, C., Tsoulfas, P., Blackmore, M.G., Lee, F.S., Liston, C., Morishita, H., and Petsko, G. A. (2020). mGreenLantern: a bright monomeric fluorescent protein with rapid expression and cell filling properties for neuronal imaging. Proc. Natl. Acad. Sci. USA *117*, 30710–30721. https://doi.org/10.1073/pnas.2000942117.

39. White, J.J., and Sillitoe, R.V. (2013). Development of the cerebellum: From gene expression patterns to circuit maps. Wiley Interdiscip. Rev. Dev. Biol. *2*, 149–164. https://doi.org/10.1002/wdev.65.

40. Sotelo, C. (2004). Cellular and genetic regulation of the development of the cerebellar system. Prog. Neurobiol. *72*, 295–339. https://doi.org/10.1016/j.pneurobio.2004.03.004.

41. Chizhikov, V.V., and Millen, K.J. (2020). Neurogenesis in the cerebellum. In Patterning and Cell Type Specification in the Developing CNS and PNS (Elsevier), pp. 349–367. https://doi.org/10.1016/B978-0-12-814405-3.00016-3.

42. Namba, K., Sugihara, I., and Hashimoto, M. (2011). Close correlation between the birth date of purkinje cells and the longitudinal compartmentalization of the mouse adult cerebellum. J. Comp. Neurol. *519*, 2594–2614. https://doi.org/10.1002/cne.22640.

43. Fujita, H., Aoki, H., Ajioka, I., Yamazaki, M., Abe, M., Oh-Nishi, A., Sakimura, K., and Sugihara, I. (2014). Detailed expression pattern of Aldolase C (Aldoc) in the cerebellum, retina and other areas of the CNS studied in Aldoc-Venus knock-in mice. PLoS One *9*, e86679–27. https://doi.org/10.1371/journal.pone.0086679.

44. Gravel, C., Eisenman, L.M., Sasseville, R., and Hawkes, R. (1987). Parasagittal organization of the rat cerebellar cortex: Direct correlation between antigenic purkinje cell bands revealed by mabQ113 and the organization of the olivocerebellar projection. J. Comp. Neurol. *265*, 294–310. https://doi.org/10.1002/cne.902650211.

45. Susaki, E.A., Shimizu, C., Kuno, A., Tainaka, K., Li, X., Nishi, K., Morishima, K., Ono, H., Ode, K.L., Saeki, Y., et al. (2020). Versatile whole-organ/body staining and imaging based on electrolyte-gel properties of biological tissues. Nat. Commun. *11*, 1982. https://doi.org/10.1038/s41467-020-15906-5.

46. Romanowski, A.J., Richardson, R.R., Plachez, C., Erzurumlu, R.S., and Poulopoulos, A. (2025). Gene Knockout in the Developing Brain of Wild-Type Rodents by CRISPR In Utero Electroporation. Methods Mol. Biol. *2910*, 221–238. https://doi.org/10.1007/978-1-0716-4446-1_13.

47. Robertson, C.D., Davis, P., Richardson, R.R., Iffland, P.H., Vieira, D.C.O., Steyert, M., McKeon, P.N., Romanowski, A.J., Crutcher, G., Jašarević, E., et al. (2023). Rapid modeling of an ultra-rare epilepsy variant in wild-type mice by in utero prime editing. Preprint at bioRxiv *12*. https://doi.org/10.1101/2023.12.06.570164.

48. Duportet, X., Wroblewska, L., Guye, P., Li, Y., Eyquem, J., Rieders, J., Rimchala, T., Batt, G., and Weiss, R. (2014). A platform for rapid prototyping of synthetic gene networks in mammalian cells. Nucleic Acids Res. *42*, 13440–13451. https://doi.org/10.1093/nar/gku1082.

49. Richardson, R.R., Steyert, M., Khim, S.N., Crutcher, G.W., Brandenburg, C., Robertson, C.D., Romanowski, A.J., Inen, J., Altas, B., and Poulopoulos, A. (2023). Enhancing Precision and Efficiency of Cas9-Mediated Knockin Through Combinatorial Fusions of DNA Repair Proteins. CRISPR J. *6*, 447–461. https://doi.org/10.1089/crispr.2023.0036.

50. Brandenburg, C., and Poulopoulos, A. (2025). Sparse Labeling, Rapid Clearing, and Native Fluorescence Light Sheet Imaging in the Developing Rodent Cerebellum. In Synapse Development: Methods and Protocols, T. Soykan and A. Poulopoulos, eds. (Springer US), pp. 263–276. https://doi.org/10.1007/978-1-0716-4446-1_16.

51. Stroh, T., Erben, U., Kühl, A.A., Zeitz, M., and Siegmund, B. (2010). Combined pulse electroporation–a novel strategy for highly efficient transfection of human and mouse cells. PLoS One *5*, e9488. https://doi.org/10.1371/journal.pone.0009488.

52. Blatt, G.J., Brandenburg, C., and Poulopoulos, A. (2023). India ink to 3D imaging: The legacy of Dr. Deepak "Dee" N. Pandya and his influence on generations of neuroanatomists. The Journal of comparative neurology *531*, 1875–1882. https://doi.org/10.1002/cne.25551.

STAR★METHODS

KEY RESOURCES TABLE

REAGENT or RESOURCE	SOURCE	IDENTIFIER
**Bacterial and virus strains**		
One shot TOP10 chemically competent E. coli	Invitrogen	C404003
**Chemicals, peptides, and recombinant proteins**		
Paraformaldehyde (PFA)	Sigma Aldrich	441244
Cubic-L	Fisher Scientific	T3740
Cubic-R+(M)	Fisher Scientific	T3741
**Critical commercial assays**		
NEB Golden Gate Assembly Kit	New England Biolabs	E1602
Zyppy Plasmid Miniprep Kit	Zymo Research	D4036
ZymoPURE II Plasmid Midiprep Kit	Zymo Research	D4200
**Experimental models: Organisms/strains**		
CD1(ICR)	Charles River	022
AldocV, Zebrin II-Venus, C57BL/6N	Comprehensive Brain Science Network	AldocV, RBRC11898, RIKEN BRC; Aldoc-Venus:C57BL/6N, 5620954, TMDU Mice Key Bank; MGI:5620954,[43]
**Recombinant DNA**		
pCAG_eGFP; µ027	This paper	N/A
mScarlet-floxed-stop-mGreenLantern–dudCas9>pJ2; ∧612	This paper	N/A
pCAG_Cre; @333	Poulopoulos et al. 2024[15]	N/A
mScarlet-floxed-stop-mGreenLantern>pJ2; ∧603	This paper	N/A
mGreenLantern-floxed-stop-mScarlet>pJ2; ∧604	This paper	N/A
GPI-mScarlet-floxed-stop-GPI-mGreenLantern>pJ2; ∧631	This paper	N/A
Cas9–Cre>pS23-CAG; ∧624	This paper	N/A
Myr-tdTomato–H2B-GFP>pCAG; @736	Trichas et al. 2008	Addgene plasmid #26771
**Software and algorithms**		
Arivis	Zeiss	https://www.zeiss.com/microscopy/us/l/campaigns/software-overview.html
Microsoft Excel	Microsoft	https://products.office.com/de-de/excel
NIS Elements	Nikon	https://www.microscope.healthcare.nikon.com/products/software/nis-elements
ImageJ	NIH	https://imagej.net
Zen Pro	Zeiss	https://www.zeiss.com/microscopy/us/products/software/zeiss-zen.html

EXPERIMENTAL MODEL AND STUDY PARTICIPANT DETAILS

Electroporation and EM experiments were performed on outbred CD1 mice, purchased timed pregnant from Charles River Laboratories to be delivered at least one day before surgery, performed across a range of ages (e.g., E11.5, E14.5, adult). Mice referred to as Zebrin II-YFP correspond to adult heterozygous Aldoc-Venus knock-in line (AldocV[43]) on a C57BL/6N background. Animals were housed in an animal facility with free access to food and water on a 12/12 h light/dark cycle. All experiments involving animal procedures were approved by the Institutional Care and Use Committees (IACUC) of the University of Maryland School of Medicine (AUP-0720005, AUP-00000251), Baylor College of Medicine (AN-5996), and of Tokyo Medical and Dental University

(A2021-065A, A2023-119C). Embryonic day 0.5 is noon on the morning a plug is identified and P0 is the day of pup birth. Since embryos are targeted at random with *in utero* electroporation, both sexes are represented at random. Electroporated pups were kept with the dam until perfusion at various developmental ages, typically E15.5, E17.5, P0, P7 and P14.

METHOD DETAILS

Plasmids

All new plasmids were custom designed and constructed with a modified mMoClo system.[48] Parts were cloned with Bsa1 adapters and assembled into expression constructs with a NEB Golden Gate Assembly Kit (NEB #E1602). Plasmids were transformed into One Shot TOP10 (Thermo Fisher C404006) chemically competent E. Coli, grown in LB broth ~18 h at 37° and extracted with Zyppy Plasmid Miniprep kits (Zymo Research D4036) to perform diagnostic digests and Sanger sequencing (Azenta Life Sciences). ZymoPURE II Plasmid Midiprep kits (Zymo Research D4200) were used to obtain a high concentration of validated plasmids for use in IUE experiments. Plasmid details including links with sizes and sequences are provided in the key resources table, results, figures and Figure S1. All plasmids expressed fluorophores or Cre recombinase from a CAG promoter.

In utero electroporation

In utero electroporation was performed as described in,[49] with the tritrode configuration[9] to target the Purkinje cell progenitor zone at E11.5 or cortical progenitor zone at E14.5.[50] Pregnant dams were anesthetized with isoflurane and pain medications were delivered as specified by the lab's animal use protocol. Hair removal cream (Veet) was applied to remove hair from the abdomen, which was then sterilized with alcohol wipes and betadine before making a ~3 cm incision along the midline of both the skin and muscle layers. Embryos were gently pulled through the incision with a blunt forceps and consistently covered with warmed PBS with a transfer pipette throughout the surgery. 4-5μL of DNA to a maximum total concentration of 4 μg/μL was injected into the 3rd or 4th ventricle at E11.5 and into the lateral ventricle at E14.5. Plasmids with a size discrepancy were molar matched (using an online calculator) to eliminate plasmid concentration as a variable. Fluorophore plasmids were typically delivered at ~2μg/μL and Cre plasmids were titrated, generally to ~20ng/μL. A tweezertrode with both ends connected to the negative port on the electroporator was placed on the lateral sides of the embryo and a third, independent positive electrode was directed over the progenitor zone of interest. For the cerebellum, the area targeted is directly over the center/top of the 4th ventricle, where the rhombic lip is located. For the cortex, the independent electrode was typically placed over the midline, with either one or both lateral ventricles filled. Six square pulses were applied for 50 ms with an interval of 1 s at 24V for the cerebellum at E11.5 and 35V for the cortex at E14.5. Embryos were covered in PBS and returned to the abdominal cavity. Both the muscle layer and skin layer were sutured with a continuous stitch and the dam was allowed to recover on a heat pad then monitored for five days to provide appropriate analgesics. P0 pups were screened for fluorescence under a Leica stereoscope and positive pups were kept with the dam until the appropriate developmental age. As areas electroporated vary between animals, plasmid comparisons are best done within one animal. N is indicated within each figure legend. Overall, we analyzed differential plasmid labeling in $N = 3$ sectioned brains and $N = 1$ light sheet brain for the plasmid pair shown in Figure 1C and $N = 3$ sectioned brains for the plasmid pair in Figure 1E in the cortex. In the cerebellum, we analyzed $N = 10$ for the plasmid pair in Figure 1E with stereoscope imaging and $N = 1$ light sheet imaging, as well as $N = 5$ light sheet imaging with the plasmid pair in Figure 1C.

Titrating cre in EKM plasmid mixtures

With the tritrode electroporation approach, each lateral ventricle can be targeted separately with plasmid mixtures while each hemisphere is targeted with the same electrical field (over the midline of the brain). This eliminates the need to interpret differences across animals and imaging to directly compare changes in fluorophore expression. The mosaic plasmid (2 μg/μL) described in Figure 1E is co-electroporated with either a low Cre concentration (15 ng/μL) in one ventricle or a high Cre concentration (150 ng/μL) in the other ventricle. Low Cre concentrations preserve endogenous fluorophore expression of both RFP and GFP and can be titrated to produce a 50/50 ratio between colors (Figure S3). However, using excessive Cre concentrations results in a loss of the floxed RFP endogenous expression while the GFP expression is reduced. Although not confirmed, we assume that the loss in GFP expression is a result of recombination of the plasmid to nonfunctional forms from the excessive Cre being produced in most electroporated cells. Higher Cre concentrations will result in a greater likelihood of cells being coelectroporated with both the mosaic plasmid and the Cre plasmid, which leads to more cells that are GFP positive up until the point that plasmids are presumably recombined to non-functional forms. The higher the Cre concentration, the more likely it is to have low overall RFP expression because only the cells subjected to the weakest electrical pulses will receive few copies of the RFP plasmid and not any Cre plasmid.

Weak electroporation results in a lower copy number of plasmids that enter the cells and therefore appear dimmer than cells that receive higher copy numbers under a stronger electrical field.[51] This can be observed in Figure S3B where the RFP is strongest in the deeper layers and progressively fades in the upper layers while GFP expression is weak in the deeper layers and bright in the superficial layers. The combination of mosaic plasmid copy numbers that contribute to the fluorophore pool and electroporation of the Cre plasmid based on its size/concentration lead to the overall pattern of expression. Because the ratio of GFP to RFP is sensitive to small changes in Cre concentration, efforts must be made to use the same aliquots of Cre plasmid across experimental animals. Different stocks (as in Figure S3) have to be titrated to achieve the desired ratio of GFP to RFP. When one fluorophore color becomes dim due

to low copy numbers of plasmids, expression in distal processes can be lost in comparison to the brighter fluorophore. Additionally, as plasmids lose supercoiling, they effectively have less concentration of supercoiled plasmid that will be electroporated (Figure S2). Therefore, proper storage of plasmids is important and should be run undigested on a gel periodically to detect shifts in supercoiled state.

Perfusion and tissue processing

Mice were transcardially perfused with ice-cold PBS followed by 4% PFA and postfixed in cold 4% PFA for 24 h. Sections were sliced on a vibratome in cold cutting conditions at 80 μm, mounted on glass slides and coverslipped with Fluoromount mounting media (Thermo Fisher 00-4958-02) then imaged at 10x (Nikon Ti2-E inverted microscope) immediately for best retention of endogenous plasmid expression. Room temperature sections (in PBS) lose fluorescence intensity over a few hours, so must be kept cold, otherwise will need to be stained with immunofluorescence. Whole cerebellum images were taken with a Leica stereoscope directly after perfusion and before further processing to avoid high GFP background after post-fixation.

Clearing and light sheet imaging

Brains were cleared to transparency as described[52] by a modified Cubic protocol.[36] Whole PFA-fixed brains were placed into 100% Cubic-L in a shaking water bath at 37°C. Cubic-L was replaced every two days until the brain was uniformly opaque and white matter tracts were no longer seen (about one week). Brains are then briefly rinsed in Cubic-R+(M) and placed in fresh Cubic-R+(M) shaking at room temperature until transparent (about two days). Whole brain images were acquired with a Zeiss Light Sheet 7 with lenses adjusted to a refractive index of 1.52 with a 5× objective and visualized with Arivis software.

Transmission electron microscopy

Purkinje cells from the cerebellum were subjected to TEM imaging following established protocols, $N = 3$. After transcardial perfusion of Ringer's solution followed by modified Karnovski's fixative (pH 7.4), sagittal sections of the cerebellum measuring 1–2 mm in thickness were prepared at room temperature. These sections were then immersed in modified Karnovski's fixative in 0.1 M sodium cacodylate buffer at pH 7.2 and rotated in scintillation vials for a period of three days. On the third day, the tissue was processed inside a Ted Pella Bio Wave Vacuum Microwave on ice. Samples were fixed again, followed by 3x sodium cacodylate buffer rinses, post-fixed with 1% buffered osmium tetroxide, and followed again with 3 millipore water rinses. Ethanol concentrations from 30 to 100% were used as the initial dehydration series, followed with propylene oxide as a final dehydrant. The samples were gradually infiltrated with propylene oxide and Embed 812 resin, with several changes of pure resin under vacuum. Following overnight infiltration in pure resin on a rotator, the samples were embedded into regular Beem capsules and cured in an oven at 62°C for five days. After polymerization, the embedded samples were thin-sectioned to 50 nm and stained with 1% uranyl acetate for fifteen minutes, followed by lead citrate for two minutes before TEM examination. TEM imaging was performed using a JEOL JEM 1010 transmission electron microscope with an AMT XR-16 mid-mount 16 mega-pixel digital camera. Subsequent to imaging, adjustments to image contrast were made using ImageJ software.

QUANTIFICATION AND STATISTICAL ANALYSIS

The use of different plasmids within one brain allows for clear visualization of separation between plasmids so we mainly chose a qualitative approach for presenting the data described. For the light sheet images, Arivis segmentation of individual Purkinje cells was achieved with the "blob finder" analysis pipeline. Each identified cell is listed as an object, all objects found with each color (RFP and GFP) were counted then RFP was expressed as a percentage of GFP expressing cells. For quantification of layer distribution of RFP vs. GFP cells in the cortex, ROIs with the pial surface at the top of the horizontal plane were cropped and ImageJ cell counter was utilized to manually mark each cell body (RFP/GFP coexpression, GFP only or RFP only). The position of each cell along the y axis was exported, with zero as closest to the pial surface from $N = 3$ brains in each condition. Cell numbers and standard deviations are reported in figure legends.

Quantification of bubble features were manually performed for each cell by directly placing the scalebar over features of interest within each imaging modality and representing the data in the figure legends as mean and standard deviation from n = 15 myr-RFP/H2B-GFP, $n = 20$ GPI-GFP and $n = 17$ electron microscopy Purkinje cells.

Cell Reports Methods, Volume *5*

Supplemental information

Developmental transformations of Purkinje cells tracked by DNA electrokinetic mobility

Cheryl Brandenburg, Garrett W. Crutcher, Andrea J. Romanowski, Sarah G. Donofrio, Lita R. Duraine, Richard N.A. Owusu-Mensah, Benjamin H. Cooper, Izumi Sugihara, Gene J. Blatt, Roy V. Sillitoe, and Alexandros Poulopoulos

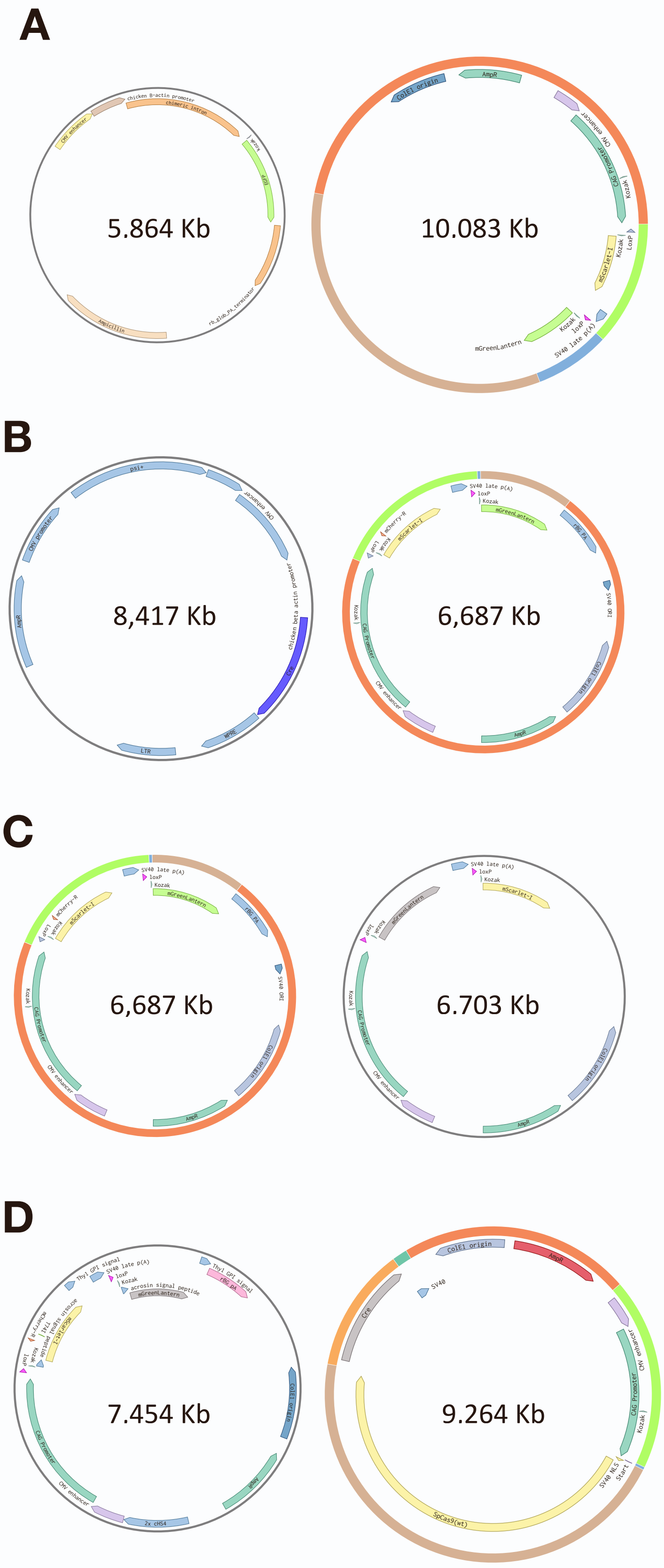

Figure S1. Construct maps and sizes of plasmid pairs used to determine the effect of electrokinetic mobility on *in utero* electroporation, related to Figure 1.
A, Fig. 1B,C; Fig. 5A; **B,** Fig. 1E, Fig. 2, Fig. 3B; **C,** Fig. 1D; **D,** Fig. 5B,D. All pairs aside from **C** are unmatched in electrokinetic mobility. Inserts located after poly A tails will not be translated so can be used to increase plasmid size without transcriptional impact. Backbones elements (as in **B**) were removed without expression consequences to decrease size and match the partner plasmid's size.

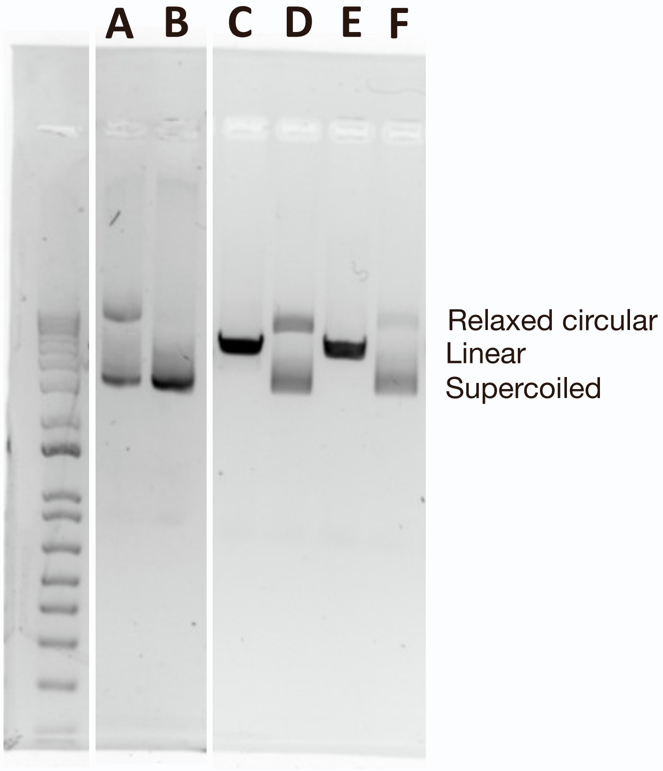

Figure S2. Plasmid electrokinetic mobility changes in gel electrophoresis, related to Figure 1. Each lane (A-F) represents the same plasmid of size 5,956 Kb, run on two different gels. Lane A was a several month old plasmid prep subjected to periods of room temperature and freeze-thaw cycles, while lane B was the same plasmid freshly prepared and frozen at -20˚C (run undigested). Lane B does not show the open circular form present in Lane A from extended storage times at higher temperatures[S1] and is entirely in the supercoiled form. The plasmid prep in lane A is poorly transfected during in utero electroporation while the lane B plasmid has high transfection rates. The different preps from lane A and B both show the same linear band when a restriction digest is performed to cut the plasmid in one location (lanes C and E). When incubated at 37˚C for one hour and then run undigested, the compromised plasmid (from lane A) further deteriorates away from the supercoiled form and toward the relaxed circular form (lane D), while the fresh prep from lane B also begins to show relaxation, but to a lesser degree (lane F). Plasmids used for in utero electroporation should be run undigested to ensure all preps maintain most of their concentration in the supercoiled form.

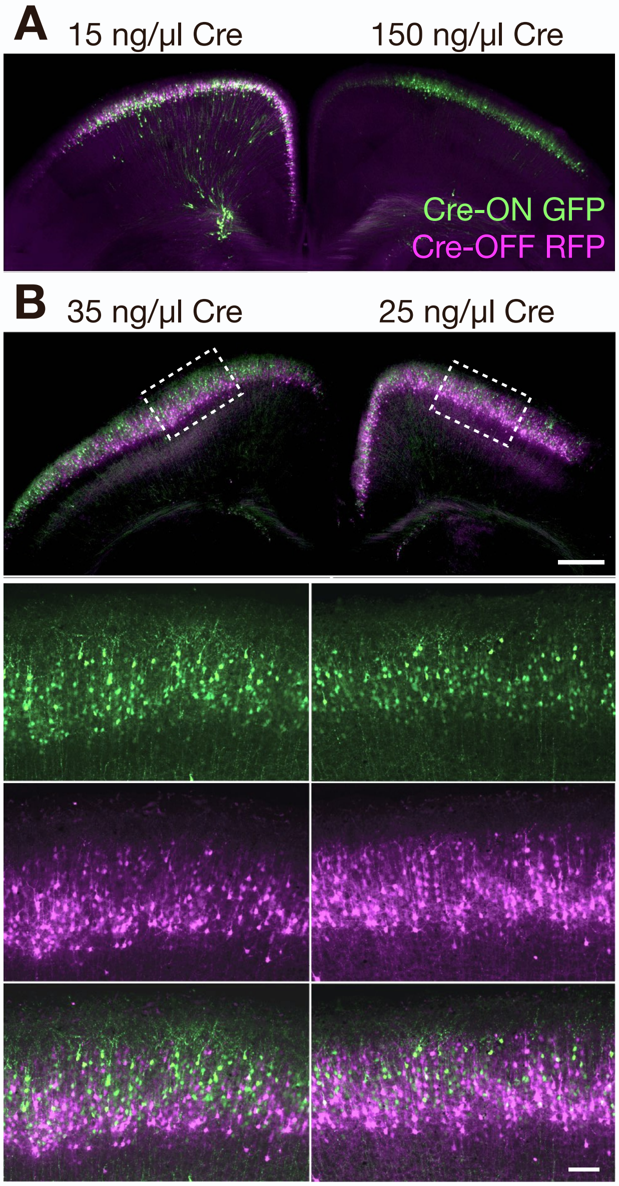

Figure S3. Titrated EKM plasmid mixtures enable "progenitor zone stereo-tracking" by differentially targeting distinct depths of the the progenitor field with one electroporation, related to Figure 1.
Each lateral ventricle in the same animal was separately injected with the Cre-dependent mosaic plasmids (plasmids b in Extended Data Fig. 1) expressing Cre-ON GFP (green) at two different concentrations of Cre plasmid (ng/μl plasmid DNA as indicated), differences in expression of the fluorophores in neuron populations and fluorescent protein intensity. **A,** Electroporation of embryos at E16.5 suppresses layer segregation since no further cortical neuron layers are produced during beyond that time point. The hemisphere that received 150 ng/μl Cre plasmid showed diminished RFP expression, indicating that most cells received Cre, which excised the RFP open reading frame. **B,** Layered labeling appear electroporation at E14.5 and earlier. Cre concentration effects layering segregation as well as and the ratio of GFP to RFP expressing cells. Cells at the ventricular surface during electroporation and migrating to superficial layers receive higher concentrations of Cre (GFP positive), while the slow Cre plasmid does not reach as many cells away from the ventricular surface at the time of electroporation, which migrate to deeper cortical layers (RFP positive). Scale bar 500 μm in main panel, 100 μm in inset.

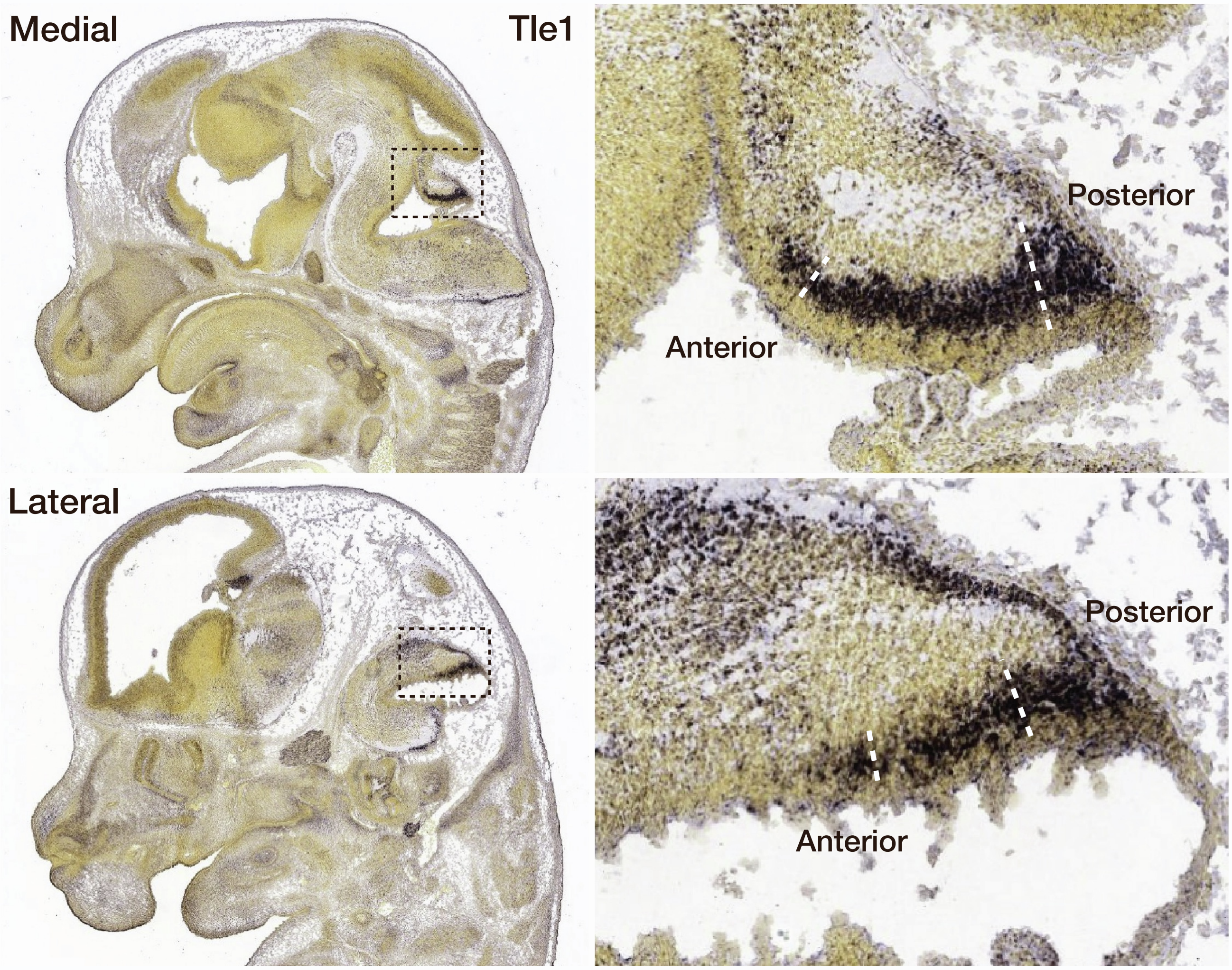

Figure S4. The Purkinje cell progenitor zone, related to Figure 4. Allen Developing Brain Atlas labeling all Purkinje cells at E13.5 as they form the Purkinje cell plate. In the cerebellum, *Tle1* is a Purkinje cell marker in early development[S2] and serial sections are provided by the Allen Institute after *in situ* hybridization with *Tle1* probe. In the medial posterior, Purkinje cell layering is thickest, which eventually becomes the vermis that is labeled most densely with RFP (Fig. 2 in main). Moving both in the anterior and lateral direction, the Purkinje cell field becomes thinner, with the least number of Purkinje cell layers across the anterior portion of the ventricular zone (in the dorsal to ventral plane of layering). A larger proportion anterior cells are close to the ventricular surface at the time of the electroporation thus mainly become GFP expressing (Fig. 3J in main for the shape of the cerebellar anlage).

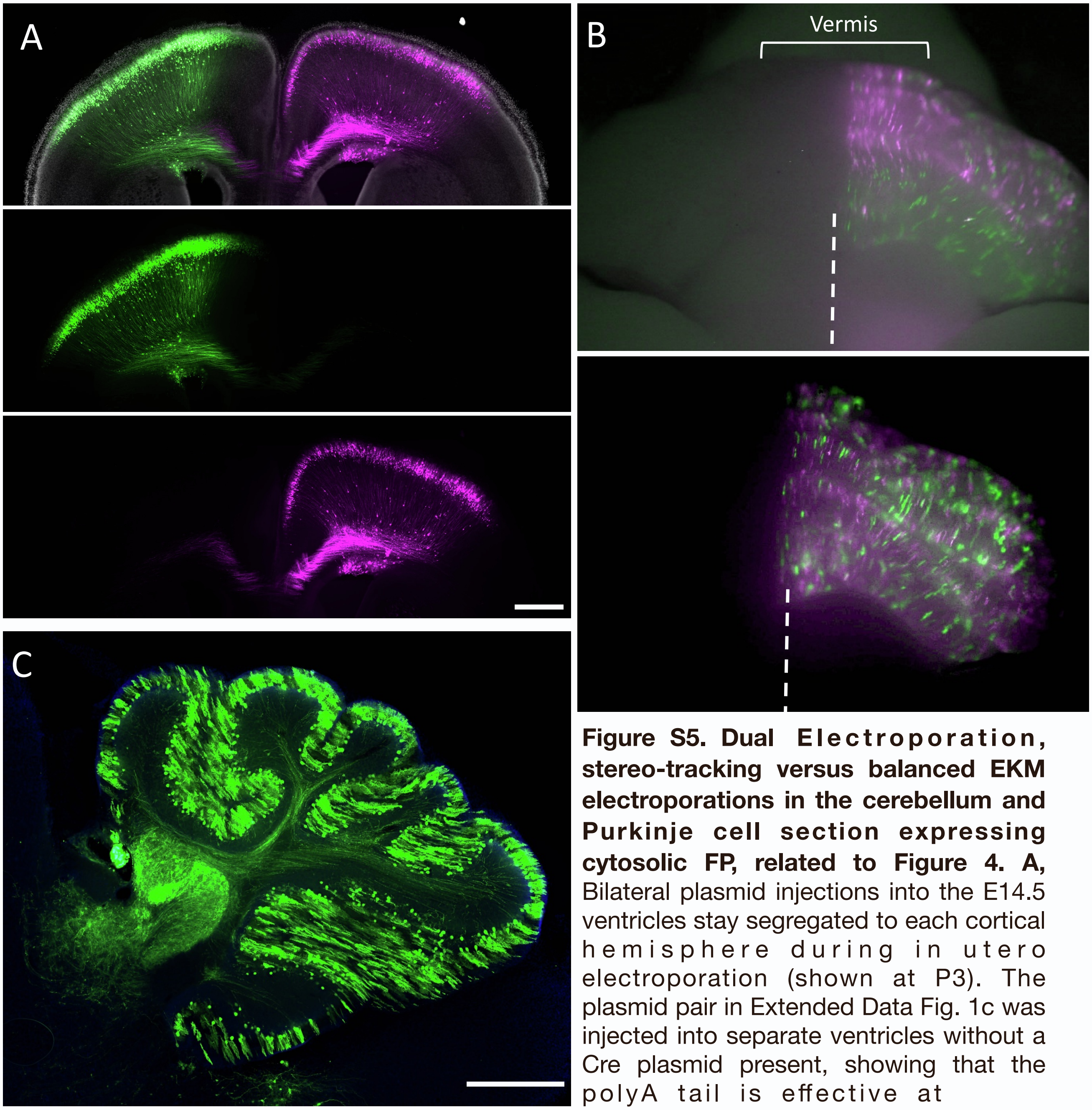

Figure S5. Dual Electroporation, stereo-tracking versus balanced EKM electroporations in the cerebellum and Purkinje cell section expressing cytosolic FP, related to Figure 4. A, Bilateral plasmid injections into the E14.5 ventricles stay segregated to each cortical hemisphere during in utero electroporation (shown at P3). The plasmid pair in Extended Data Fig. 1c was injected into separate ventricles without a Cre plasmid present, showing that the polyA tail is effective at preventing translation of the second fluorophore. B**,** P14 cerebella after electroporation at E11.5 using stereoscope whole-mount images. Figure 3C (left image) of the main text shows segregation between the two colors of stereo-tracking plasmids. The bright area of RFP expression (magenta) in the vermis is eliminated when size-matched plasmids are utilized (right image) and the anterior lobules now have equal representation of RFP and GFP (green). Therefore, when plasmid pairs no longer have a size differential (right image), the electroporation has equal distribution and segregation effects are no longer apparent. **C,** Cytosolic fluorophores more brightly label cell bodies. Simultaneously imaging both axons and cell bodies results in saturated cell bodies but dim axons. This presents a particular obstacle with 3D light sheet imaging where bright cell body fluorescence scatters in the unlabeled cleared tissue increasing background. Additionally, cytosolic FPs do not label axon bubbles. Scale bars 500 µm or stereoscope image.

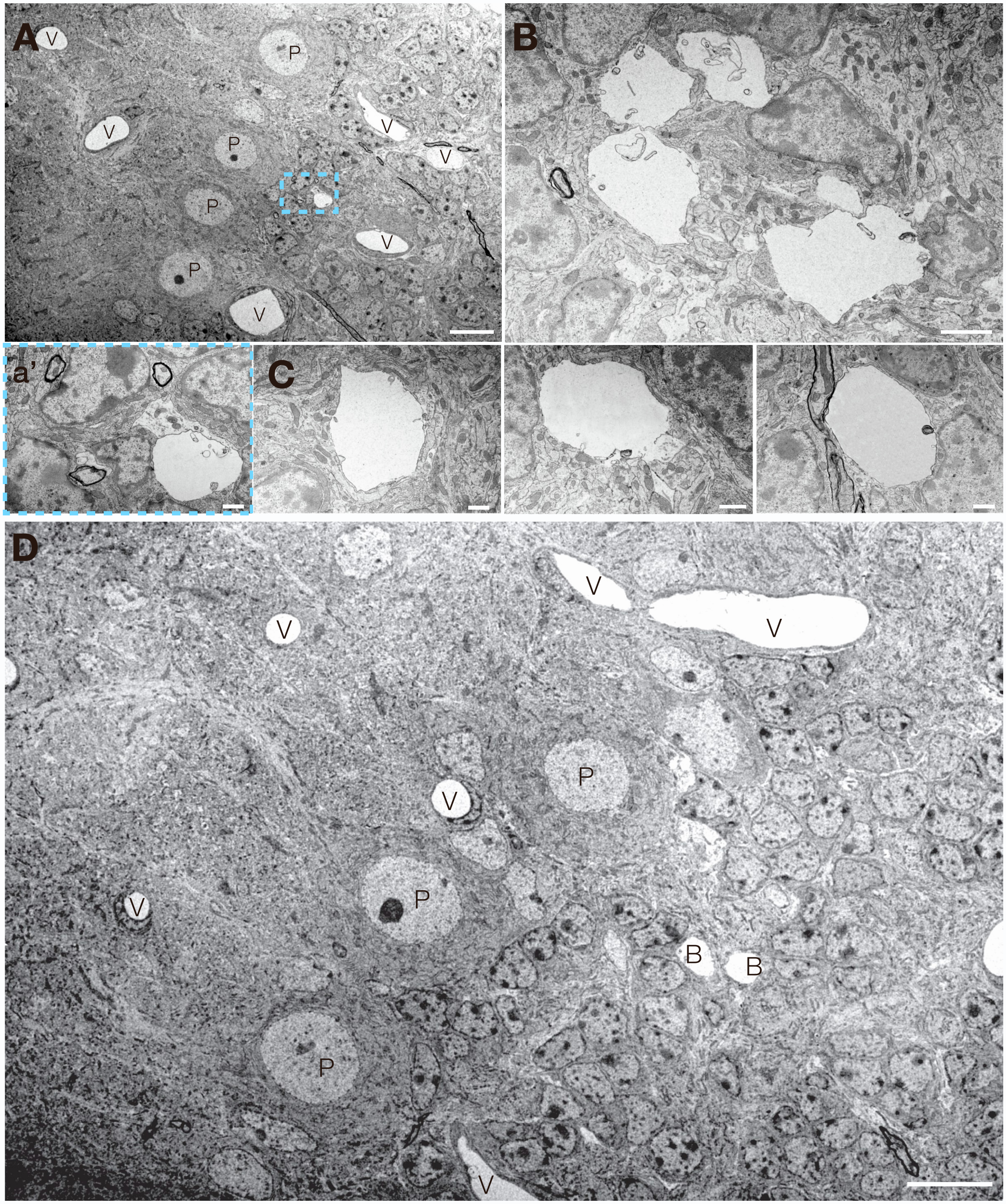

Figure S6. Examples of electrolucent bubbles in electron micrographs, related to Figure 5. A, Electron micrographs of Purkinje cell (P) layer showing an overview of the axon bubble shown on the right panel of Fig. 5E. Electrolucent axon bubbles appear sporadically about 10 µm from the Purkinje cell soma on the granule cell layer side, which are not surrounded by endothelial cells like blood vessels (V) seen throughout the tissue. The area outlined in dashed blue, containing the axon bubble, is magnified in α'. Scale bar 10 µm, 1 µm inset. **B-C**) Higher magnification of individual axon bubbles highlights their irregular shapes, lack of endothelial cells/pericytes, and position along structures associated with the axon. Scale bars 2 µm (B) and 1 µm (C). **D,** Overview showing electrolucent structures scattered throughout the tissue.

The majority are blood vessels (V), clearly identified by vascular cells forming a dense surrounding dark layer, however, axon bubbles (B) sporadically appear 6-30um from the Purkinje cell body (P) within the granule cell layer only. They are not found elsewhere in the tissue and can be differentiated from the blood vessels since they are not surrounded by vascular cells. Scale bar 10 μm.

SUPPLEMENTARY REFERENCES

S1. Schleef, M., Baier, R., Walther, W., Michel, M.-L., and Schmeer, M. (2006). Long-Term Stability Study and Topology Analysis of Plasmid DNA By Capillary Gel Electrophoresis. BioProcess Int *4*.

S2. Wizeman, J.W., Guo, Q., Wilion, E.M., and Li, J.Y.H. (2019). Specification of diverse cell types during early neurogenesis of the mouse cerebellum. eLife *8*, 1–24. https://doi.org/10.7554/eLife.42388.
